# Taeanamides A and B, Nonribosomal Lipo-Decapeptides Isolated from an Intertidal-Mudflat-Derived *Streptomyces* sp.

**DOI:** 10.3390/md20060400

**Published:** 2022-06-16

**Authors:** Jinsheng Cui, Eunji Kim, Dong Hyun Moon, Tae Ho Kim, Ilnam Kang, Yeonjung Lim, Daniel Shin, Sunghoon Hwang, Young Eun Du, Myoung Chong Song, Munhyung Bae, Jang-Cheon Cho, Jichan Jang, Sang Kook Lee, Yeo Joon Yoon, Dong-Chan Oh

**Affiliations:** 1 Natural Products Research Institute, College of Pharmacy, Seoul National University, Seoul 08826, Korea; cuijs@snu.ac.kr (J.C.); eunjikim@snu.ac.kr (E.K.); mdh931022@gmail.com (D.H.M.); pharmcraft87@gmail.com (D.S.); sunghooi@snu.ac.kr (S.H.); dye0302@snu.ac.kr (Y.E.D.); smch517@snu.ac.kr (M.C.S.); sklee61@snu.ac.kr (S.K.L.); 2 Molecular Mechanism of Antibiotics, Division of Life Science, Department of Bio & Medical Big Data (BK4 Program), Research Institute of Life Science, Gyeongsang National University, Jinju 52828, Korea; taeho12349@gmail.com (T.H.K.); jichanjang@gnu.ac.kr (J.J.); 3 Department of Biological Sciences, Inha University, Incheon 22212, Korea; ikang@inha.ac.kr (I.K.); yj_lim@inha.ac.kr (Y.L.); chojc@inha.ac.kr (J.-C.C.); 4 College of Pharmacy, Gachon University, Incheon 21936, Korea; baemoon89@gachon.ac.kr

**Keywords:** intertidal mudflat, *Streptomyces* sp., anti-tuberculosis, cytotoxicity, nonribosomal peptide synthetase

## Abstract

Two new lipo-decapeptides, namely taeanamides A and B (**1** and **2**), were discovered from the Gram-positive bacterium *Streptomyces* sp. AMD43, which was isolated from a mudflat sample from Anmyeondo, Korea. The exact molecular masses of **1** and **2** were revealed by high-resolution mass spectrometry, and the planar structures of **1** and **2** were elucidated using NMR spectroscopy. The absolute configurations of **1** and **2** were determined using a combined analysis of ^1^H-^1^H coupling constants and ROESY correlations, the advanced Marfey’s method, and bioinformatics. The putative nonribosomal peptide synthetase pathway for the taeanamides was identified by analyzing the full genome sequence data of *Streptomyces* sp. AMD43. We also found that taeanamide A exhibited mild anti-tuberculosis bioactivity, whereas taeanamide B showed significant bioactivity against several cancer cell lines.

## 1. Introduction

Nonribosomal peptides (NRPs) are structurally diverse peptide molecules produced by multi-modular nonribosomal peptide synthetases (NRPSs) [1], and they have been shown to exhibit a wide variety of biological activities, such as antibiotic and anticancer properties, in addition to acting as immunosuppressive agents [2,3]. Because of their structural and biological diversities, the exploration of new classes of NRPs could be an effective approach to discovering novel bioactive chemical scaffolds for pharmaceutical and medicinal applications. The investigation of marine-derived actinomycetes, which have been recently highlighted as a source of bioactive organic compounds [4], was found to yield a number of NRPs. For example, thiocoraline, which was isolated from a marine actinobacterial strain belonging to the genus *Micromonospora*, entered a preclinical trial as an anticancer drug candidate [5,6]. In our group, the search for new bioactive NRPs has focused on marine-derived microorganisms and has continuously provided new NRP classes with novel features in their structures and biosynthetic pathways. For example, the chemical analysis of *Streptomyces* sp. SNJ042 collected from a sandy beach led to the discovery of an antitubercular and anticancer cyclic depsipeptide, ohmyungsamycin A, which contained a methoxy tryptophan and a β-hydroxy phenylalanine residues [7]. In addition, structurally novel pseudodimeric peptides, mohangamides A and B, were discovered from an intertidal mudflat *Streptomyces* sp. SNM55 and were reported to act as *Candida albicans* isocitrate lyase inhibitors [8]. Deeper analysis of the metabolites and the genomic sequence of the SNM55 strain elucidated unprecedented noncanonical features in terms of the biosynthesis of WS9326A [9]. Furthermore, our co-cultivation of marine *Streptomyces* and *Bacillus* strains induced the production of a new cyclic hexapeptide, dentigerumycin E, and we elucidated its NRP biosynthetic pathway along with its antiproliferative and antimetastatic activities against human cancer cell lines [10].

**Figure 1:**
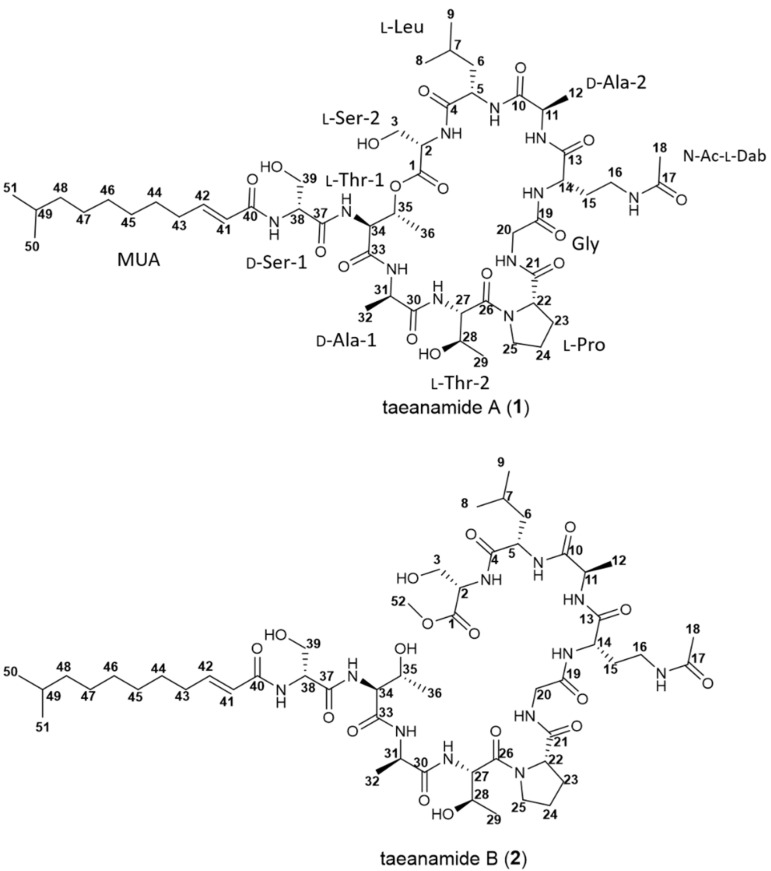
Structures of taeanamides A (**1**) and B (**2**).

In this context, we herein report our investigation into two new NRPs, taeanamides A and B (**1** and **2**) (Figure 1). More specifically, following the selective isolation of actinobacterial strains from a marine mudflat on Anmyeondo on the west coast of the Republic of Korea, the chemical profile of the *Streptomyces* sp. AMD43 strain was examined by LC/MS analysis to detect a couple of previously unreported molecules displaying a common UV absorption maximum at 225 nm along with pseudomolecular ions at *m*/*z* 1108 and 1122, respectively. Further scale-up and subsequent purification enabled us to characterize the compounds spectroscopically and elucidate their structures. We herein report the structural elucidation, biological evaluation, and biosynthetic pathways of these new NRPs.

## 2. Results and Discussion

Taeanamide A (**1**) was isolated as a white powder with a molecular formula of C_51_H_85_N_11_O_16_ as determined by ^1^H and ^13^C NMR data together with HRESIMS data (obsd. [M + H]^+^ at *m*/*z* 1108.6240, calcd 1108.6249). This molecular formula indicated 15 degrees of unsaturation in **1**. By analyzing the ^1^H NMR and multiplicity-edited HSQC NMR data of **1** (in DMSO-*d*_6_), a total of 10 amide protons (δ_H_ 8.33–7.67), a set of vicinal olefinic protons (δ_H_ 6.65 and 6.06, *J* = 15.0 Hz), 21 protons directly connected to heteroatom-bound carbons (δ_H_ 5.34–3.03), 20 methylene protons and 2 methine aliphatic protons (δ_H_ 2.14–1.13), and 9 methyl groups (δ_H_ 1.79, 1.23, 1.22, 1.08, 1.02, 0.84 (6H, overlapped), 0.82, and 0.80) were identified (Figures S1 and S4), indicating that taeanamide A is a modified peptide that incorporates with a *trans*-olefin-bearing lipophilic moiety. Consistently, analysis of the ^13^C NMR spectrum along with the HSQC spectrum identified 12 carbonyl groups (δ_C_ 172.5, 172.1, 171.5, 171.4 (2C), 171.0, 169.8, 169.3, 169.2, 168.4 (2C), and 165.4), two olefinic carbons (δ_C_ 143.5 and 123.8), 16 N/O-bound carbons (δ_C_ 70.2–35.5), and 21 alkyl carbons (δ_C_ 41.7–16.1) (Table 1).

**Figure 2:**
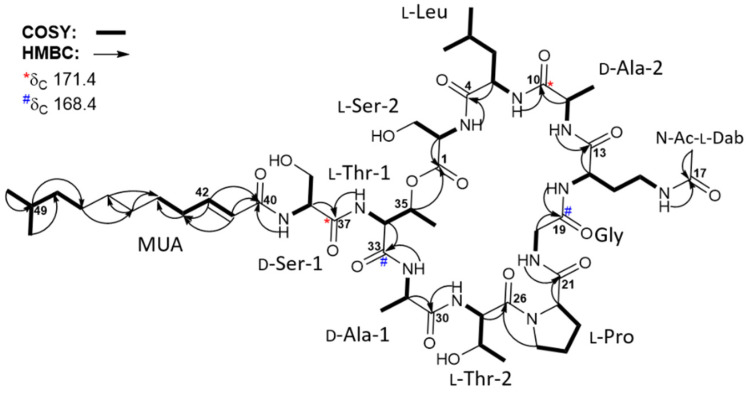
Key COSY and HMBC correlations in taeanamide A (**1**). Overlapped carbonyl carbons at δ_C_ 171.4 and 168.4 are noted.

All one-bond carbon–proton correlations were assigned by interpreting the HSQC spectrum together with the ^1^H and ^13^C NMR data (Table 1). Analysis of the COSY, TOCSY, and HMBC NMR spectra revealed 10 amino acid residues, including nine proteinogenic amino acids (two serine, two threonine, two alanine, proline, glycine, and leucine) and one unusual amino acid (*N*(4)-acetyl-2,4-diaminobutyric acid) (Figure 2). In detail, COSY and TOCSY correlations among the 14-NH (δ_H_ 8.15), H-14 (δ_H_ 4.19), H_2_-15 (δ_H_ 1.77 and 1.64), H_2_-16 (δ_H_ 3.03), and 16-NH (δ_H_ 7.81) revealed a 14-NH—C-14—C-15—C-16—16-NH connectivity. Furthermore, HMBC correlations from H-14 (δ_H_ 4.19) to C-13 (δ_C_ 171.0) and from 16-NH (δ_H_ 7.81) and H_2_-16 (δ_H_ 3.03) to C-17 (δ_C_ 169.2) confirmed the locations of C-17 and 16-NH. Moreover, the H_3_-18 methyl protons (δ_H_ 1.79) exhibited an HMBC correlation with C-17, thereby allowing elucidation of the partial structure of *N*(4)-acetyl-2,4-diaminobutyric acid (*N*-Ac-Dab) (Figure 2).

The lipophilic moiety was also identified by analyzing the COSY and HMBC spectra. Initially, the COSY correlations of H-41 (δ_H_ 6.06)/H-42 (δ_H_ 6.65), H-42 (δ_H_ 6.65)/H-43 (δ_H_ 2.14), and H-43 (δ_H_ 2.14)/H-44 (δ_H_ 1.38) revealed a carbon connection of C-41–C-42–C-43–C-44. Similarly, the C-47–C-48–C-49–C-50(C-51) partial structure was assigned based on the H-47 (δ_H_ 1.24)/H-48 (δ_H_ 1.13), H-48 (δ_H_ 1.13)/H-49 (δ_H_ 1.50), H-49 (δ_H_ 1.50)/H_3_-50 (δ_H_ 0.84), and H-49 (δ_H_ 1.50)/H_3_-51 (δ_H_ 0.84) COSY correlations. Due to the fact that the chemical shifts of the H-45, H-46, and H-47 protons overlapped at 1.24–1.27 ppm, HMBC correlations rather than COSY correlations were utilized to extend the partial structures. Thus, the HMBC correlations of H-49 (δ_H_ 1.50)/C-47 (δ_C_ 26.7), H-47 (δ_H_ 1.24)/C-45 (δ_C_ 28.6), H-45 (δ_H_ 1.27)/C-46 (δ_C_ 29.1), H-43 (δ_H_ 2.14)/C-44 (δ_C_ 27.8), and H-46 (δ_H_ 1.25)/C-44 (δ_C_ 27.8) allowed the C-44–C-45–C-46–C-47 connectivity to be determined (Figure 2). Furthermore, the HMBC correlations of H-41 (δ_H_ 6.06)/C-40 (δ_C_ 165.4) and H-42 (δ_H_ 6.65)/C-40 (δ_C_ 165.4) confirmed the location of C-40 next to C-41, thereby revealing the lipophilic chain to consist of 10-methylundec-2-enoic acid (MUA) (Figure 2).

**Figure 3:**
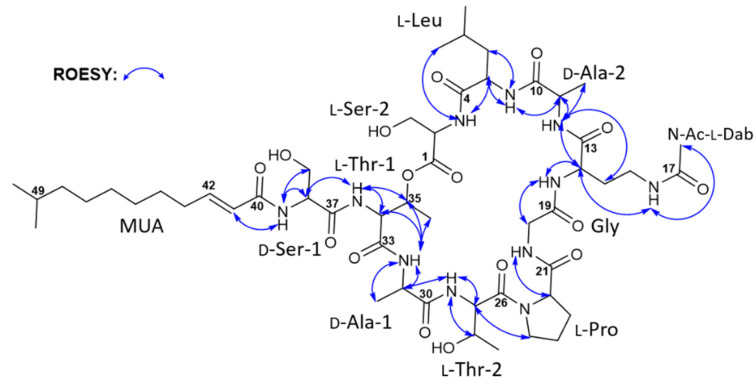
Key ROESY correlations in taeanamide A (**1**).

The elucidated partial structures were initially assembled based on the HMBC correlations (Figure 2). However, the overlapped carbonyl resonances (two carbons at δ_C_ 171.4 and two carbons at δ_C_ 168.4) hampered the unequivocal determination of the sequence of the subunits, resulting in the utilization of ROESY correlations (Figure 3) together with HMBC correlations. 2-NH (δ_H_ 8.18)/C-4 (δ_C_ 171.8) HMBC correlation revealed the linkage from Ser-2 to Leu. This connectivity was confirmed by the ROESY correlation from 2-NH (δ_H_ 8.18) to H-5 (δ_H_ 4.51) and H_3_-8 (δ_H_ 0.80). 11-NH (δ_H_ 8.33)/C-13 (δ_C_ 171.0) correlation established connectivity between Ala-2 and *N*-Ac-Dab, which was consistent with 11-NH (δ_H_ 8.33)/H-14 (δ_H_ 4.19) and 11-NH (δ_H_ 8.33)/H_2_-15 (δ_H_ 1.77 and 1.64). 20-NH (δ_H_ 7.67)/C-21 (δ_C_ 171.5) HMBC correlation assigned the Gly residue immediately adjacent to Pro. Thr-2 was also located beside the Pro residue, as determined based on the H-25b (δ_H_ 3.59)/C-26 (δ_C_ 169.8) three-bond HMBC correlation. Furthermore, the 27-NH (δ_H_ 8.08)/C-30 (δ_C_ 172.1) correlation confirmed the connection between Thr-2 and Ala-1. These assignments were also supported by 20-NH (δ_H_ 7.67)/H-22 (δ_H_ 4.26), H-25b (δ_H_ 3.59)/H-27 (δ_H_ 4.53), and 27-NH (δ_H_ 8.08)/H-31 (δ_H_ 4.28) ROESY couplings. The acyl chain, MUA, was then connected to Ser-1 based on the HMBC correlation from the distinct olefinic proton H-41 (δ_H_ 6.06) and 38-NH (δ_H_ 8.17) to the separated carbonyl carbon C-40 at δ_C_ 165.4. Then, four fragmental structures, MUA—Ser-1, Ala-1—Thr-2—Pro—Gly, *N*-Ac-Dab—Ala-2, and Leu—Ser-2, were constructed, and four carbonyl carbons, two carbons at δ_C_ 171.4 and two carbons at δ_C_ 168.4, were not yet assigned. Two carbons at δ_C_ 171.4 displayed heteronuclear correlations with H-11 (δ_H_ 4.22), H-38 (δ_H_ 4.50), 11-NH (δ_H_ 8.33), and 34-NH (δ_H_ 8.26). H-11 and H-38 were α-carbons of Ala-2, and Ser-2, assigning these two carbonyl carbons at δ_C_ 171.4 as C-10, and C-37 in Ala-2, and Ser-2, respectively. 5-NH (δ_H_ 7.67)/H-11 (δ_H_ 4.22) was observed to connect Leu and Ala-2 while 34-NH (δ_H_ 8.26)/H-38 (δ_H_ 4.50) ROESY correlation deciphered the linkage between Ser-1 and Thr-1. Therefore, the fragments were further assembled as *N*-Ac-Dab—Ala-2—Leu—Ser-2 and MUA—Ser-1—Thr-1. The two carbons overlapped at δ_C_ 168.4 correlated with 14-NH (δ_H_ 8.15), H-20a (δ_H_ 3.85), 31-NH (δ_H_ 7.76), and H-34 (δ_H_ 4.43), allocating these carbons as the carbonyl carbons of Gly and Thr-1. Gly was inserted between *N*-Ac-Dab and Pro by 14-NH (δ_H_ 8.15)/H-20a (δ_H_ 3.85) and 20-NH (δ_H_ 7.67)/H-22 (δ_H_ 4.26) ROESY correlations, extending the peptide chain to make Ala-1—Thr-2—Pro—Gly—*N*-Ac-Dab—Ala-2—Leu—Ser-2. The Thr-1 unit was assigned in the middle of Ser-1 and Ala-1 based on 34-NH (δ_H_ 8.26)/H-38 (δ_H_ 4.50), 31-NH (δ_H_ 7.76)/H-34 (δ_H_ 4.43), and 31-NH/H-35 (δ_H_ 5.34) ROESY correlations. Thus, the sequence of the lipopeptide was elucidated as MUA—Ser-1—Thr-1—Ala-1—Thr-2—Pro—Gly—*N*-Ac-Dab—Ala-2—Leu—Ser-2. Furthermore, the characteristically deshielded proton H-35 (δ_H_ 5.34) indicated the existence of an ester bond. By observed HMBC correlation of H-35 (δ_H_ 5.34)/C-1 (δ_C_ 169.3), the ester linkage between C-1 and C-35 was confirmed. In addition, by qTOF-HR-MS/MS analysis of taeanamide A (**1**), the observed MS/MS fragments provided additional evidence to support the elucidated amino acid sequence of taeanamide A (**1**) (Figure S16). Consequently, taeanamide A (**1**) was identified as a new cyclic lipo-decapeptide containing *N*-Ac-Dab and MUA as unusual units.

The geometric configuration of the C-41—C-42 double bond was determined to be 41*E* by the analysis of ^3^*J*_H41H42_ coupling constant (15.0 Hz), while the absolute configurations of the amino acids present in **1** were determined by applying the advanced Marfey’s method [11]. More specifically, following the acidic hydrolysis of **1**, the hydrolysate was separately derivatized with l- and d-1-fluoro-2,4-dinitrophenyl-5-alanine amide (l- and d-FDAA), and these FDAA derivatives were analyzed by LC/MS. The elution orders of the l- and d-FDAA derivatives revealed the absolute configurations of amino acids in **1** to be d-Ala, l-Thr, l-Leu, and l-Pro. However, the absolute configurations of the two Ser residues remained undetermined since one Ser appeared to possess the l-configuration, whereas the other exhibited the d-configuration (Table S1). In addition, during acidic hydrolysis, *N*-Ac-Dab is converted to 2,4-diaminobutyric acid (Dab). Although the l-FDAA di-adduct of Dab eluted faster than its d-FDAA derivative, the absolute configuration of this unit could not be determined because predicting the elution order of the two advanced Marfey products (i.e., the l-and d-di-FDAA adducts) was not straightforward. We then derivatized authentic l-Dab with l- and d-FDAA [12], and analysis of the product retention times revealed the elution of l- to d-FDAA di-adducts (Table S3). Due to the fact that the Marfey adducts of the Dab unit from **1** eluted at retention times consistent with those of l-Dab, the absolute configuration of *N*-Ac-Dab was established to be l. Furthermore, the additional chiral center of the β-position in Thr was assigned as *R* based on the elution order of the d-FDAA derivative of Thr in **1** and the d-FDAA products of standard l-Thr and l-*allo*-Thr, thereby indicating that taeanamide A (**1**) contains the l-Thr residue.

Taeanamide B (**2**) was also purified as a white powder, and its molecular formula was confirmed by HRFABMS (obsd. [M + H-H_2_O]^+^ at *m*/*z* 1122.6400, calcd 1122.6405) to have the molecular formula C_52_H_89_N_11_O_17_. The 1D and 2D NMR data of taeanamide B indicated that it possessed a structure highly similar to that of taeanamide A. Comprehensive comparison of the NMR data revealed that taeanamide B contains an additional methoxy group (δ_H_ 3.63; δ_C_ 52.5). Further analysis of the HMBC spectrum assigned a methoxy group at the C-terminus based on the H_3_-52/C-1 correlation. All the exchangeable protons (including hydroxyl groups) of taeanamide B were shown in ^1^H-NMR, which proved taeanamide B to be an acylic lipo-decapeptide. The absolute configuration of taeanamide B was also determined using the advanced Marfey’s method in the same manner as described for **1**, and it was found that taeanamides A and B share the same absolute configurations in their amino acid units (Table S2). This result is further supported by the identical CD spectra of **1** and **2** (Figure S15). The nature of the acyclic methyl ester of **2** might indicate that taeanamide B (**2**) is an artifact of **1** by using MeOH in the purification procedure. However, taeanamide B initially existed in the EtOAc extract, and taeanamide A (**2**) was not converted into **2** in its MeOH solution at room temperature during 10 days (Figure S19). Therefore, it is proposed that taeanamide B was also produced as a natural product from *Streptomyces* sp. AMD43.

**Figure 4:**
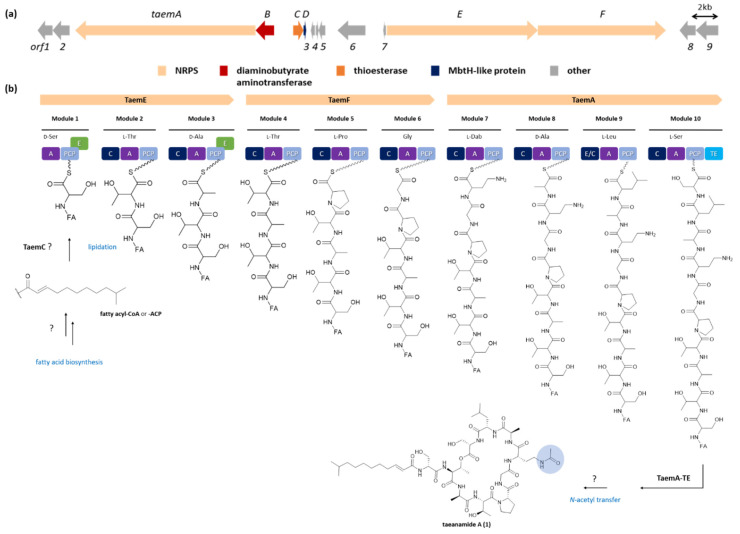
Proposed biosyntheses of taeanamides A and B. (**a**) Genetic organization of the *taem* gene cluster. (**b**) The activated fatty acyl chain is transferred to Ser-activating module 1 in the early stages of biosynthesis, and then three NRPSs (TaemE, TaemF, and TaemA) build up the peptide chain prior to release of the mature chain. Further modifications to yield taeanamide A and B are carried out by unascertained catalytic enzymes that are not encoded within the taeanamide biosynthetic gene region. A, adenylation domain; C, condensation domain; E, epimerization domain; E/C, dual epimerization and condensation domain; PCP, peptidyl carrier protein domain; and TE, thioesterase domain.

As in the case of taeanamide A, the absolute stereochemistries of the two Ser residues could not be determined for taeanamide B, and therefore bioinformatic analysis of the biosynthetic gene cluster of the taeanamides was required, as described below. Thus, complete structural assignments of the taeanamides were accomplished by careful analysis of the biosynthetic gene cluster. Initially, it was considered that the structures of the taeanamides suggested that their biosyntheses depend on the NRPS machinery. A candidate NRPS gene cluster was therefore identified for the taeanamides through bioinformatics analysis of the genome sequence of *Streptomyces* sp. AMD43 using antiSMASH 5.0 software (Table S4) [13]. Three clustered NRPS genes termed *taemE* (modules 1, 2, and 3), *taemF* (modules 4, 5, and 6), and *taemA* (modules 7, 8, 9, and 10) were predicted to be responsible for the biosynthesis of the taeanamide peptide backbone (Figure 4). Three additional genes, encoding a diaminobutyrate aminotransferase, a thioesterase, and an MbtH-like protein, appeared to be related to the gene cluster found upstream of the *taemA* gene (Figure 4a).

Each Taem NRPS module comprises condensation (C), adenylation (A), and peptidyl carrier protein (PCP) domains. Two additional epimerization (E) domains and a thioesterase (TE) domain are located in modules 1, 3, and 10, respectively (Figure 4b). According to the Stachelhaus prediction [14], the predicted substrate specificities of the A domains in the taeanamide NRPSs were not consistent with all amino acid building blocks (Table S5). Although the A domains of modules 3 and 8 were predicted to activate Ser, Ala should also have been incorporated into these modules. Similarly, Dab and Leu were incorporated in modules 7 and 9, respectively, contrary to what was predicted, i.e., activated Gln and Val. It should be noted here that when the substrate-specific code of the A domain showed 80% less agreement with the Stachelhaus code, differences were observed between the predicted and actual amino acids in the taeanamides (Table S5).

Through amino acid sequence alignment and phylogenetic analysis of C domains within the Taem NRPSs and other lipopeptide synthetases (Figures S17 and S18), the Taem C domains were categorized into three distinct groups. Particularly, the C3, C5, C6, C7, C8, and C10 domains were assigned as ^L^C_L_ domains catalyzing peptide bond formation between two l-amino acids, while the C2 and C4 domains were categorized as ^D^C_L_ domains linking an l-amino acid to a C-terminal d-amino acid of the growing peptide chain [15]. It could therefore be expected that the two amino acid residues, Ser and Ala, which were incorporated into modules 1 and 3, respectively, are d-form amino acids whose incorporation is catalyzed by conventional E domains that convert the l-amino acids into their d-isomers [16]. Furthermore, the eighth C domain in module 9 (TaemA-C9) was identified as a dual epimerization and condensation (E/C) domain [15,17]. It should be noted here that all C domains possess a conserved core motif (motif 3, HHxxxDG), while the E/C domains harbor an additional HH[I/L]xxxxGD motif at their N-terminus. This dual E/C domain of module 9 was predicted to contribute to isomerization of the C-terminal l-Ala residue of the elongated PCP-bound peptidyl intermediate located in module 8, followed by condensation to the downstream aminoacyl extender unit. This combination of genetic analysis and Marfey’s method therefore allowed the configurational assignment of the Ser and Ala residues as d-Ser-1, d-Ala-1, d-Ala-2, and l-Ser-2 (Figure 1 and Figure 4).

As shown in Figure 4b, Taem synthetase contains a type I thioesterase (TE) domain (TaemA-TE) at the C-terminus of the last module that catalyzes the release and cyclization of the taeanamide backbone. An external type II TE-encoding protein (TaemC) was assumed to resume the installed biosynthesis by removing aberrant intermediates or regenerating misprimed carrier proteins [18]. It was also considered that TaemC may be involved in the initiation of taeanamide biosynthesis by transferring the fatty acyl chain to the Ser-activating module 1, as has been verified in the type II TE SrfD of surfactin biosynthesis [19]. Although there are a number of ways to incorporate a fatty acid moiety into the PCP-tethered aminoacyl thioester of the first module, a starter C (Cs) domain located in the *N*-terminus of the NRP assembly line is usually responsible for catalyzing such mechanisms [20]. However, we were unable to find any Cs or Cs-like domains within Taem synthetase based on our phylogenetic analysis of the C domains (Figure S18), nor were any genes involved in fatty acid activation and attachment found in the gene cluster. It was therefore expected that TaemC may participate in this unclear lipidation reaction associated with *trans*-fatty acid activation from other biosynthesis pathways (Figure 4b).

The amino acid incorporated by module 7 was predicted to be an l-Dab formed from aspartate β-semialdehyde by the TaemB homolog of PvdH, which encodes a diaminobutyrate aminotransferase from pyoverdine biosynthesis [21]. This assumption was made since *taemB* was the only gene that we found to be related to modification of the taeanamide gene cluster. Further *N*-acetyl transfer of the l-Dab residue to promote conversion to *N*-Ac-l-Dab is likely conducted by other biosynthetic routes. One possibility is that *N*-Ac- l-Dab is obtained from the biosynthesis of ectoine [22], since three ectoine gene clusters exist in the AMD43 genome. Likewise, the addition of a methyl group while making an acyclic peptide for taeanamide B conversion may be catalyzed by a methyltransferase homolog encoded by a gene from outside the taeanamide gene cluster (Figure 4b).

Subsequently, the cytotoxicities of **1** and **2** were evaluated against several human cancer cell lines. Although taeanamide A (**1**) showed no cytotoxic activity (IC_50_ > 20 μM), taeanamide B (**2**) strongly inhibited human lung cancer (A549), colon cancer (HCT116), breast cancer (MCF-7, MDA-MB-231), liver cancer (SK-HEP-1), and stomach cancer (SNU638) cell lines (IC_50_ = 0.26–1.13 μM) (Table 2). Considering the structural difference between taeanamides A and B, it was apparent that the linear form (**2**) provides much higher cytotoxicity than the cyclized form (**1**).

Finally, antibacterial and antifungal assays were carried out, and it was found that taeanamides A and B (**1** and **2**) were mostly inactive against the tested pathogenic bacteria (i.e., *Staphylococcus aureus, Enterococcus faecalis, Enterococcus faecium, Klebsiella pneumoniae, Salmonella enterica,* and *Escherichia coli*) and fungi (i.e., *Candida albicans, Aspergillus fumigatus, Trichophyton rubrum,* and *Trichophyton mentagrophytes*). However, taeanamides A and B exhibited a moderate inhibition of *Mycobacterium tuberculosis* mc^2^ 6230. In this assay, taeanamide A displayed the highest antitubercular activity of the two compounds with an MIC_50_ of 27 ± 0.03 μM, whereas taeanamide B showed a relatively reduced effect with an MIC_50_ of 63 ± 0.05 μM (bedaquiline as the positive control, MIC_50_ = 0.4 ± 0.01 μM).

## 3. Materials and Methods

### 3.1. General Experimental Procedures

Optical rotations were measured using a Jasco P-2000 polarimeter (Tokyo, Japan) with a 1.0 cm cell. The UV spectra were recorded using a Perkin Elmer Lambda 35 UV/VIS spectrometer (Waltham, MA, USA). Circular dichroism (CD) measurements were carried out using an Applied Photophysics Ltd. Chirascan-Plus CD detector (Leatherhead, UK), while the infrared (IR) spectra were acquired using a JASCO FT/IR-4200 spectrometer (Tokyo, Japan). High-resolution fast atom bombardment mass spectroscopy (HRFABMS) was carried out using a JEOL JMS-700 high-resolution mass spectrometer (Tokyo, Japan) at the National Center for Inter-University Research Facilities (NCIRF). High-resolution electrospray ionization (ESI) LC/MS data were obtained using an AB SCIEX Q-TOF 5600 high-resolution mass spectrometer (Framingham, MA, USA) at the National Instrumentation Center for Environmental Management (NICEM, Seoul, South Korea). qTOF-HR-MS/MS analysis was performed on a Waters XEVO^®^ G2S Q-TOF mass spectrometer (Milford, MA, USA). The ^1^H, ^13^C, and 2D NMR spectra were recorded using Bruker Avance 800 MHz NMR spectrometer (Billerica, MA, USA) at the College of Pharmacy, Seoul National University. Low-resolution electrospray ionization (ESI) LC/MS data were collected on an Agilent Technologies 6130 quadrupole mass spectrometer (Santa Clara, CA, USA) coupled with an Agilent Technologies 1200 series HPLC using a reversed-phase C_18_(2) column (Phenomenex Luna, 5 μm, 4.6 × 100 mm^2^).

### 3.2. Isolation and Identification of the Bacterial Strain Streptomyces sp. AMD43

The *Streptomyces* sp. AMD43 strain was isolated from an intertidal mudflat sample collected in Anmyeondo, Republic of Korea. Various isolation media were used to isolate the actinobacterial strains, and the AMD43 strain was isolated from YEME agar medium (10 g malt extract, 4 g yeast extract, 4 g glucose, 18 g agar, and 27 g sea-salt per 1 L of deionized water), which was incubated at 27.5 °C for 1 week. The AMD43 strain (GenBank accession number OM319619) was found to be most closely related to *Streptomyces europaeiscabiei* KACC 20186 (99% identity, GenBank accession number #NR_042790.1), based on 16S rRNA gene sequence analysis.

### 3.3. Cultivation and Extraction

The *Streptomyces* sp. AMD43 strain was inoculated on YEME agar medium plates and grown at 28 °C for 1 week. The bacterial spores on the plates were transferred into YEME liquid medium (50 mL) in a 125 mL flask and incubated at 28 °C under 200 rpm rotation for 4 d. An aliquot (10 mL) of the AMD43 liquid seed culture was then inoculated into the YEME liquid medium (200 mL) in a 500 mL flask. After cultivation at 160 rpm for 4 d, an aliquot (20 mL) of the liquid culture was inoculated in YEME liquid medium (1 L) in a 2.8 L Fernbach flask, and the culture was maintained for 6 d. After the incubation, the whole culture liquid sample was extracted with ethyl acetate (EtOAc) using a separatory funnel. The residual water was then removed from the EtOAc layer by the addition of anhydrous sodium sulfate. After filtration, the organic material was concentrated in vacuo to yield a dry extract. In total, 72 L of the liquid culture was cultivated and extracted.

### 3.4. Isolation of Taeanamides A (***1***) and B (***2***)

The obtained extract was dissolved in methanol (MeOH) and divided into three equal parts. Each fraction was fractionated using a Sephadex LH-20 column (15 × 550 mm, mobile phase: MeOH), wherein the fractions containing the taeanamides were eluted at 70–80 min (flow rate: 0.8 mL/min). Taeanamides A and B were further purified using reversed-phase HPLC (Kromasil column 100-5-C_18_, 10 × 250 mm, 45% aqueous CH_3_CN isocratic conditions, detection: UV 210 nm, flow rate: 2 mL/min) to obtain taeanamide A (60 mg) and taeanamide B (90 mg) at 19 and 28 min, respectively.

Taeanamide A (**1**): White powder, [α]_D_ +40.4 (c 0.1, MeOH); UV (MeOH) λ_max_ (log *ε*) 225 (3.71) nm; IR (neat) ν_max_ 3316, 1657, 1536 cm^−1^; for ^1^H and ^13^C NMR data, see Table 1, HRFABMS *m*/*z* 1130.6112 [M + Na]^+^ (calcd for C_51_H_85_N_11_O_16_Na, 1130.6074), HRESIMS *m*/*z* 1108.6240 [M + H]^+^ (calcd for C_51_H_86_N_11_O_16_, 1108.6249).

Taeanamide B (**2**): White powder, [α]_D_ +34.6 (c 0.1, MeOH); UV (MeOH) λ_max_ (log *ε*) 225 (3.70) nm; IR (neat) ν_max_ 3312, 1657, 1538 cm^−1^; for ^1^H and ^13^C NMR data, see Table 1, HRFABMS *m*/*z* 1122.6400 [M + H-H_2_O]^+^ (calcd for C_52_H_88_N_11_O_16_, 1122.6405).

### 3.5. qTOF-HR-MS/MS Analysis of Taeanamides A (***1***) and B (***2***)

Taeanamides A and B solution for MS analysis was prepared at 300 μg/mL concentration in 50% *aq*. CH_3_CN (0.1% formic acid). A direct sample injection at a flow rate of 20 μL/min was applied. The MS system was operated in ESI with a positive ionization mode. The typical operating parameters were as follows: analyzer, resolution mode; capillary voltage (volt.), 3.0 kV; sampling cone volt., 30 V; source temperature (temp.), 120 °C, source offset temp., 80 °C; desolvation temp., 400 °C; cone gas flow, 10 L/h; desolvation gas flow, 600 L/h: helium collision gas. The analyzer was operated with an extended dynamic range at 60,000 resolution (FWHM at *m*/*z* 556.2771) with an acquisition time of 0.1 s. Leucine enkephalin (400 pg/μL, 50% *aq*. CH_3_CN with 0.1% formic acid) as a lockspray was infused at a rate of 5 μL/min for mass correction. Mass spectra were acquired with a scan range of 50–1200 amu with scan time 1 s. The optimized collision energies of taeanamide A and B were applied as 50 eV and 85 eV, respectively.

### 3.6. The Advanced Marfey’s Method

Taeanamide A (**1**, 3 mg) was hydrolyzed in a 6 N aqueous HCl solution (1 mL) at 115 °C with stirring for 2 h. Then the reaction vial was cooled in an ice bath for 5 min, and the reaction mixture was dried in vacuo to remove the HCl. The reaction mixture was then re-dissolved in water (1 mL) and the solution was re-dried in vacuo. This process was repeated three times to completely remove residual HCl. The hydrolysate mixture of free amino acids was then divided into two vials. To each vial was added NaHCO_3_ (0.3 mL) and 200 μL of either l-FDAA (1-fluoro-2,4-dinitrophenyl-5-l-alanine amide) or d-FDAA (10 mg/mL in acetone). The reaction mixture was then heated at 80 °C in a water bath for 5 min and subsequently quenched by the addition of a 2 N aqueous HCl solution (0.15 mL) to each vial. After drying the products in vacuo, the dried materials were dissolved in MeOH. Each sample was analyzed by LC/MS under gradient flow conditions (UV detection: 340 nm; flow rate: 0.7 mL/min; 10–60% aqueous CH_3_CN with 0.1% formic acid over 40 min) using a reversed-phase column (Phenomenex Luna C_18_ (2), 100 × 4.6 mm, 5 μm). To determine the stereochemistry of the Thr and *N*-Ac-Dab residues, authentic l-Thr, l-*allo*-Thr, and l-Dab were derivatized and analyzed using the same procedure (Table S1). The absolute configuration of taeanamide B (**2**) was analyzed using the same methods as those applied for taeanamide A (**1**) (Table S2).

### 3.7. Cytotoxicity Assay

To explore the biological activities of taeanamides A and B (**1** and **2**), their cytotoxic activities were evaluated against various human cancer cell lines, including human lung cancer (A549), colon cancer (HCT116), breast cancer (MCF-7, MDA-MB-231), liver cancer (SK-HEP-1), and stomach cancer (SNU638) cell lines. The cytotoxicity of each was measured using the sulforhodamine B (SRB) assay. Cells were seeded in 96-well plates and incubated for 30 min (for the zero-day controls) or treated with the desired compound for 72 h. After incubation, the cells were fixed, dried, and stained with 0.4% SRB in 1% acetic acid. The unbound dye was removed by washing, and the stained cells were suspended in 10 mM Tris buffer (pH 10.0). The absorbance was measured at 515 nm, and the cytotoxicity was determined. The IC_50_ values were calculated by nonlinear regression analysis using TableCurve 2D v5.01 software (Systant Software Inc., Richmond, CA, USA) [23].

### 3.8. Resazurin Microtiter Assay (REMA) Plate Testing of Mycobacterium Tuberculosis

The MIC values for taeanamide A and B against *M. tuberculosis* were determined using the REMA. For this purpose, the avirulent *M. tuberculosis* mc^2^ 6230 strain was grown at 37 °C in Middlebrook 7H9 broth (Difco) supplemented with 10% ADS (50 g/L bovine albumin, 20 g/L dextrose, and 8.5 g/L NaCl), 0.2% glycerol, 24 μg/mL pantothenate, 0.2% casamino acids, and 0.05% Tween-80. For drug susceptibility testing, aliquots (100 μL) of the medium were added to all wells of a 96-well microtiter plate, and the final bacterial OD_600_ was adjusted to 0.005. Taeanamides A and B were added to each well using a 2-fold serial dilution method. Bedaquiline was purchased from Adooq Bioscience (#A12327) and was used as a reference compound. The plates were then incubated at 37 °C for 5 d. After this time, resazurin was added to each well (0.025% (*w/v*)), and the fluorescence value was read at 560–590 nm using a SpectraMax M3 multi-mode microplate reader (Molecular Devices, CA, USA). The concentrations required to inhibit bacterial growth by 50% (IC_50_) were determined by fitting the curves using GraphPad Prism 6 software (GraphPad Software, Inc., La Jolla, CA, USA).

### 3.9. Sequencing and Gene Annotation of Streptomyces sp. AMD43

Whole-genome sequence data of the taeanamide-producing *Streptomyces* sp. AMD43 were acquired by DNA Link, Inc., using the Illumina NovaSeq 6000 platform (San Diego, CA, USA) and single-molecule sequencing technology with the PacBio RS II system (Seoul, Korea). A hybrid de novo assembly using both PacBio and Illumina data was constructed using Unicycler (v0.4.8) [24] to give a genome sequence comprising 12 contigs with a total length of 10,205,350 base pairs and a genomic GC content of 70.89%. The genome sequence was annotated with ChunLab’s in-house pipeline, which utilizes EggNOG 4.5, Swiss-Prot, KEGG, and SEED for functional annotation of the predicted protein sequences. The antiSMASH version 5.1.2 software program was utilized to predict the biosynthetic gene clusters encoded in the genome sequence, including that of the taeanamides. The genomic sequence data were deposited in GenBank under accession numbers OM307649–OM307659 and OM324356–OM324359.

## 4. Conclusions

Two new nonribosomal lipo-decapeptides, taeanamides A and B (**1** and **2,** respectively), were discovered to be present in intertidal-mudflat-derived *Streptomyces* sp. Although several cyclic lipopeptides including iturine A [25], bacillomycin D [26], mycosubtilin [27], and surfactin [28] and acyclic peptides including hoshinoamides A and B [29] and acycliclaxaphycin A [30] were discovered from nature, the structures of these taeanamides are particularly unique, with no comparable peptide natural products having been previously reported. In particular, the unusual amino acid, *N*-Ac-Dab, is extremely rare in nature. Although a comprehensive literature search found that the Dab unit is present in a selection of peptide-derived bacterial compounds, such as antibacterial cyclic peptides, permetin A [31], and circulocins [32] from *Bacillus circulans*, in addition to LPS inhibitors and pedopeptins from *Pedobacter* sp. [33], the presence of *N*-Ac-Dab has only been reported in the antibiotic diterpenoid, platensiamide A from *Streptomyces platensis* [34]. Genomic analysis of the producer *Streptomyces* sp. AMD43 revealed a putative taeanamide nonribosomal peptide synthetase (NRPS) biosynthetic gene cluster employing a dual epimerization and condensation (E/C) domain. In terms of their biological activities, taeanamide A was found to exhibit a mild antitubercular activity, whereas taeanamide B displayed potent cytotoxicity against a number of cancer cell lines. The discovery of these unique NRPSs indicates that the chemical investigation of actinobacteria inhabiting marine environments, such as intertidal mudflats, could be an effective strategy to search for structurally novel bioactive compounds.

## Figures and Tables

**Table 1:** NMR data for taeanamide A (**1**) in DMSO-*d*_6_ and taeanamide B (**2**) in pyridine-*d*_5_.

Taeanamide A (1)			Taeanamide B (2)			Taeanamide A (1)			Taeanamide B (2)		
C/H	δ_C_, Type	δ_H_, Mult (*J* in Hz)	C/H	δ_C_, Type	δ_H_, Mult (*J* in Hz)	C/H	δ_C_, Type	δ_H_, Mult (*J* in Hz)	C/H	δ_C_, Type	δ_H_, Mult (*J* in Hz)
1	169.3, C		1	172.5, C		26	169.8, C		26	171.7, C	
2	54.2, CH	4.50, m	2	56.7, CH	5.11, m	27	55.3, CH	4.53, m	27	57.8, CH	5.22, (dd, 8.5, 4.5)
2-NH		8.18, m	2-NH		9.35, (d, 6.5)	27-NH		8.08, (d, 8.5)	27-NH		8.55, (d, 8.5)
3a	61.4, CH_2_	3.89, (dd, 11.0, 4.5)	3a	63.0, CH_2_	4.36. m	28	66.5, CH	3.92, m	28	68.5, CH	4.58, m
3b		3.62, m	3b		4.22, m	28-OH		-	28-OH		6.49, (d, 5.0)
3-OH		-	3-OH		6.84, (t, 5.5)	29	19.3, CH_3_	1.02, (d, 6.5)	29	20.8, CH_3_	1.58, (d, 6.5)
4	171.8, C		4	174.3, C		30	172.1, C		30	174.1, C	
5	50.3, CH	4.51, m	5	52.6, CH	5.25, m	31	48.8, CH	4.28, m	31	51.0, CH	5.03, m
5-NH		7.67, (d, 8.0)	5-NH		8.84, (d, 9.5)	31-NH		7.76, (d, 6.5)	31-NH		9.07, (d, 6.5)
6	41.7, CH_2_	1.41, m	6	41.9, CH_2_	2.02, m	32	17.9, CH_3_	1.23, (d, 7.0)	32	18.7, CH_3_	1.70, (d, 7.0)
7	23.8, CH	1.53, m	7	25.4, CH	1.94, m	33	168.4, C		33	172.5, C	
8	21.3, CH_3_	0.82, (d, 6.5)	8	23.9, CH_3_	0.90, (d, 6.5)	34	55.7, CH	4.43, (dd, 9.0, 2.5)	34	60.6, CH	5.06, m
9	23.3, CH_3_	0.80, (d, 6.5)	9	19.8, CH_3_	0.83, (d, 6.5)	34-NH		8.26, (d, 9.0)	34-NH		9.05, (d, 8.0)
10	171.4, C		10	174.0, C		35	70.2, CH	5.34, m	35	67.8, CH	4.92, m
11	48.6, CH	4.22, m	11	50.7, CH	5.04, m	35-OH		-	35-OH		6.53, (d, 3.5)
11-NH		8.33, (d, 7.0)	11-NH		9.41, (d, 6.5)	36	16.1, CH_3_	1.08, (d, 6.5)	36	20.8, CH_3_	1.48, (d, 6.5)
12	18.0, CH_3_	1.22, (d, 7.0)	12	18.7, CH_3_	1.70, (d, 7.0)	37	171.4, C		37	173.1, C	
13	171.0, C		13	173.6, C		38	55.7, CH	4.50, m	38	58.2, CH	5.32, (dd, 12.0, 6.0)
14	51.1, CH	4.19, m	14	52.9, CH	4.97, m	38-NH		8.17, m	38-NH		9.22, (d, 6.0)
14-NH		8.15, (d, 6.5)	14-NH		8.67, (d, 7.0)	39	61.4, CH_2_	3.63, m	39a	63.3, CH_2_	4.46, m
15a	31.2, CH_2_	1.77, m	15a	32.8, CH_2_	2.59, (td, 13.5, 6.5)				39b		4.36, m
15b		1.64, (td, 14.0, 7.5)	15b		2.35, (td, 13.5, 7.0)	39-OH		-	39-OH		7.00, (t, 5.5)
16	35.5, CH_2_	3.03, m	16a	37.3, CH_2_	3.85, m	40	165.4, C		40	167.6, C	
			16b		3.55, m	41	123.8, CH	6.06, (d, 15.0)	41	124.9, CH	6.35, (d, 15.5)
16-NH		7.81, (t, 5.5)	16-NH		8.58, (t, 6.0)	42	143.5, CH	6.65, (dt, 15.0, 7.0)	42	145.4, CH	7.15, m
17	169.2, C		17	171.5, C		43	31.2, CH_2_	2.14, (dd, 14.0, 7.0)	43	32.7, CH_2_	2.09, m
18	22.6, CH_3_	1.79, m	18	23.6, CH_3_	2.01, s	44	27.8, CH_2_	1.38, m	44	29.1, CH_2_	1.30, m
19	168.4, C		19	170.8, C		45	28.6, CH_2_	1.27, m	45	30.0, CH_2_	1.19, m
20a	41.9, CH_2_	3.85, (dd, 17.0, 6.0)	20a	44.3, CH_2_	4.40, m	46	29.1, CH_2_	1.25, m	46	30.5, CH_2_	1.15, m
20b		3.55, (dd, 17.0, 3.5)	20b		4.04, (dd, 17.0, 5.0)	47	26.7, CH_2_	1.24, m	47a	37.3, CH_2_	1.02, m
20-NH		7.67, (d, 8.0)	20-NH		9.25, (t, 6.0)				47b		1.22, m
21	171.5, C		21	174.0, C		48	38.5, CH_2_	1.13, m	48	30.2, CH_2_	1.30, m
22	60.3, CH	4.26, m	22	62.3, CH	4.69, (t, 7.5)	49	27.4, CH	1.50, m	49	35.1, CH	1.24, m
23a	29.0, CH_2_	1.98, m	23	30.2, CH_2_	2.15, m	50	22.5, CH_3_	0.84, (d, 6.5)	50	12.1, CH_3_	0.86, (d, 7.5)
23b		1.78, m				51	22.5, CH_3_	0.84, (d, 6.5)	51	22.1, CH_3_	0.83, (d, 7.5)
24a	24.3, CH_2_	1.79, m	24	26.1, CH_2_	1.94, m				52	52.5, CH_3_	3.63, s
24b		1.73, m									
25a	46.8, CH_2_	3.43, m	25	48.8, CH_2_	3.79, m						
25b		3.59, m									

^1^H 800 MHz, ^13^C 200 MHz.

**Table 2:** Cytotoxicities of taeanamides A (**1**) and B (**2**) against the selected cancer cell lines.

IC_50_ (μM)	A549	HCT116	MCF-7	MDA-MB-231	SK-Hep-1	SNU638
1	>20	>20	>20	>20	>20	>20
2	0.60 ± 0.29	0.26 ± 0.07	1.13 ± 0.74	0.8 ± 0.69	0.67 ± 0.66	0.33 ± 0.02
Etoposide	0.15 ± 0.09	0.73 ± 0.15	1.43 ± 1.00	3.42 ± 3.18	2.26 ± 3.76	0.28 ± 0.19

## Data Availability

The original contributions presented in the study are included in the article/Supplementary Material; further inquiries can be directed to the corresponding author.

## Supplementary Materials

The following supporting information can be downloaded at: https://www.mdpi.com/article/10.3390/md20060400/s1, Figure S1. ^1^H NMR spectrum of taeanamide A (**1**) at 800 MHz in DMSO-*d*_6_. Figure S2. ^13^C NMR spectrum of taeanamide A (**1**) at 200 MHz in DMSO-*d*_6_. Figure S3. COSY NMR spectrum of taeanamide A (**1**) at 800 MHz in DMSO-*d*_6_. Figure S4. HSQC NMR spectrum of taeanamide A (**1**) at 800 MHz in DMSO-*d*_6_. Figure S5. HMBC NMR spectrum of taeanamide A (**1**) at 800 MHz in DMSO-*d*_6_. Figure S6. ROESY NMR spectrum of taeanamide A (**1**) at 800 MHz in DMSO-*d*_6_. Figure S7. TOCSY NMR spectrum of taeanamide A (**1**) at 800 MHz in DMSO-*d*_6_. Figure S8. ^1^H NMR spectrum of taeanamide B (**2**) at 800 MHz in pyridine-*d*_5_. Figure S9. ^13^C NMR spectrum of taeanamide B (**2**) at 200 MHz in pyridine-*d*_5_. Figure S10. COSY NMR spectrum of taeanamide B (**2**) at 800 MHz in pyridine-*d*_5_. Figure S11. HSQC NMR spectrum of taeanamide B (**2**) at 800 MHz in pyridine-*d*_5_. Figure S12. HMBC NMR spectrum of taeanamide B (**2**) at 800 MHz in pyridine-*d*_5_. Figure S13. ROESY NMR spectrum of taeanamide B (**2**) at 800 MHz in pyridine-*d*_5_. Figure S14. TOCSY NMR spectrum of taeanamide B (**2**) at 800 MHz in pyridine-*d*_5_. Figure S15. CD spectra of taeanamides A (**1**) and B (**2**). Figure S16. HR-MS/MS data of taeanamide A (**1**) and B (**2**) obtained from a Waters XEVO^®^ G2S Q-TOF mass spectrometer. Table S1. LC/MS analysis of l- and d-FDAA derivatives of the amino acids in taeanamide A (1). Table S2. LC/MS analysis of l- and d-FDAA derivatives of the amino acids in taeanamide B (2). Table S3. LC/MS analysis of l- and d-FDAA derivatives of l-2,4-diamino butanoic acid authentic sample. Table S4. Putative functions of ORFs of the taeanamides biosynthetic gene cluster in *Streptomyces* sp. AMD43. Table S5. Adenylation (A) domain substrate specificity predictions of the taeanamide NRPS. Figure S17. Multiple sequence alignment of condensation (C) domains of Taem NRPS and lipopeptide synthetases. Figure S18. Phylogenetic analysis of condensation (C) domains of Taem NRPS and lipopeptide synthetases. Figure S19. LC/MS profiles of (a) EtOAc extract of strain AMD43 showing the existence of both taeanamides A and B (**1** and **2**); (b) taeanamide A (**1**) after purification; (c) taeanamide A (**1**) after 10 days in MeOH (room temperature).
