# Supplementary material for: Taeanamides A and B, Nonribosomal Lipo-Decapeptides Isolated from an Intertidal-Mudflat-Derived Streptomyces sp
Source: Mar Drugs. 2022 Jun 16;20(6):400. doi: 10.3390/md20060400 (PMC9229766; doi:10.3390/md20060400)
Supplement: Supplementary file 1 [file marinedrugs-20-00400-s001.zip › marinedrugs-1752053-supplementary.pdf]

Supplementary Material

Taeanamides A and B, nonribosomal lipo-decapeptides isolated from an intertidal mudflat-derived *Streptomyces* sp.

Jinsheng Cui[1], Eunji Kim[1], Dong Hyun Moon[1], Tae Ho Kim[2], Ilnam Kang[3], Yeonjung Lim[3], Daniel Shin[1], Sunghoon Hwang[1], Young Eun Du[1], Myoung Chong Song[1], Munhyung Bae[4], Jang-Cheon Cho[3], Jichan Jang[2], Sang Kook Lee[1], Yeo Joon Yoon*[1], Dong-Chan Oh*[1]

[1] Natural Products Research Institute, College of Pharmacy, Seoul National University, Seoul 08826, Republic of Korea

[2] Molecular Mechanism of Antibiotics, Division of Life Science, Division of Bio & Medical Big Data Department (BK4 Program), Research Institute of Life Science, Gyeongsang National University, Jinju, Gyeongnam 52828, Republic of Korea

[3] Department of Biological Sciences, Inha University, Incheon 22212, Republic of Korea

[4] College of Pharmacy, Gachon University, Incheon 21936, Republic of Korea

*** Correspondence:**
Yeo Joon Yoon
yeojoonyoon@snu.ac.kr

Dong-Chan Oh
dongchanoh@snu.ac.kr

Table of Contents

Figure S1. ^{1}H NMR spectrum of taeanamide A (**1**) at 800 MHz in DMSO-d_6.

Figure S2. ^{13}C NMR spectrum of taeanamide A (**1**) at 200 MHz in DMSO-d_6.

Figure S3. COSY NMR spectrum of taeanamide A (**1**) at 800 MHz in DMSO-d_6.

Figure S4. HSQC NMR spectrum of taeanamide A (**1**) at 800 MHz in DMSO-d_6.

Figure S5. HMBC NMR spectrum of taeanamide A (**1**) at 800 MHz in DMSO-d_6.

Figure S6. ROESY NMR spectrum of taeanamide A (**1**) at 800 MHz in DMSO-d_6.

Figure S7. TOCSY NMR spectrum of taeanamide A (**1**) at 800 MHz in DMSO-d_6.

Figure S8. ^{1}H NMR spectrum of taeanamide B (**2**) at 800 MHz in pyridine-d_5.

Figure S9. ^{13}C NMR spectrum of taeanamide B (**2**) at 200 MHz in pyridine-d_5.

Figure S10. COSY NMR spectrum of taeanamide B (**2**) at 800 MHz in pyridine-d_5.

Figure S11. HSQC NMR spectrum of taeanamide B (**2**) at 800 MHz in pyridine-d_5.

Figure S12. HMBC NMR spectrum of taeanamide B (**2**) at 800 MHz in pyridine-d_5.

Figure S13. ROESY NMR spectrum of taeanamide B (**2**) at 800 MHz in pyridine-d_5.

Figure S14. TOCSY NMR spectrum of taeanamide B (**2**) at 800 MHz in pyridine-d_5.

Figure S15. CD spectra of taeanamides A (**1**) and B (**2**).

Figure S16. HR-MS/MS data of taeanamide A (**1**) and B (**2**) obtained from a Waters XEVO® G2S Q-TOF mass spectrometer.

Table S1. LC/MS analysis of L- and D-FDAA derivatives of the amino acids in taeanamide A (**1**).

Table S2. LC/MS analysis of L- and D-FDAA derivatives of the amino acids in taeanamide B (**2**).

Table S3. LC/MS analysis of L- and D-FDAA derivatives of L-2,4-diamino butanoic acid authentic sample.

Table S4. Putative functions of ORFs of the taeanamides biosynthetic gene cluster in *Streptomyces* sp. AMD43.

Table S5. Adenylation (A) domain substrate specificity predictions of the taeanamide NRPS.

Figure S17. Multiple sequence alignment of condensation (C) domains of Taem NRPS and lipopeptide synthetases.

Figure S18. Phylogenetic analysis of condensation (C) domains of Taem NRPS and lipopeptide synthetases.

Figures S19. LC/MS profiles of (a) EtOAc extract of strain AMD43 showing the existence of both taeanamides A and B (**1** and **2**); (b) taeanamide A (**1**) after purification; (c) taeanamide A (**1**) after 10 days in MeOH (room temperature).

Figure S1. ^{1}H NMR spectrum of taeanamide A (**1**) at 800 MHz in DMSO-d_6.

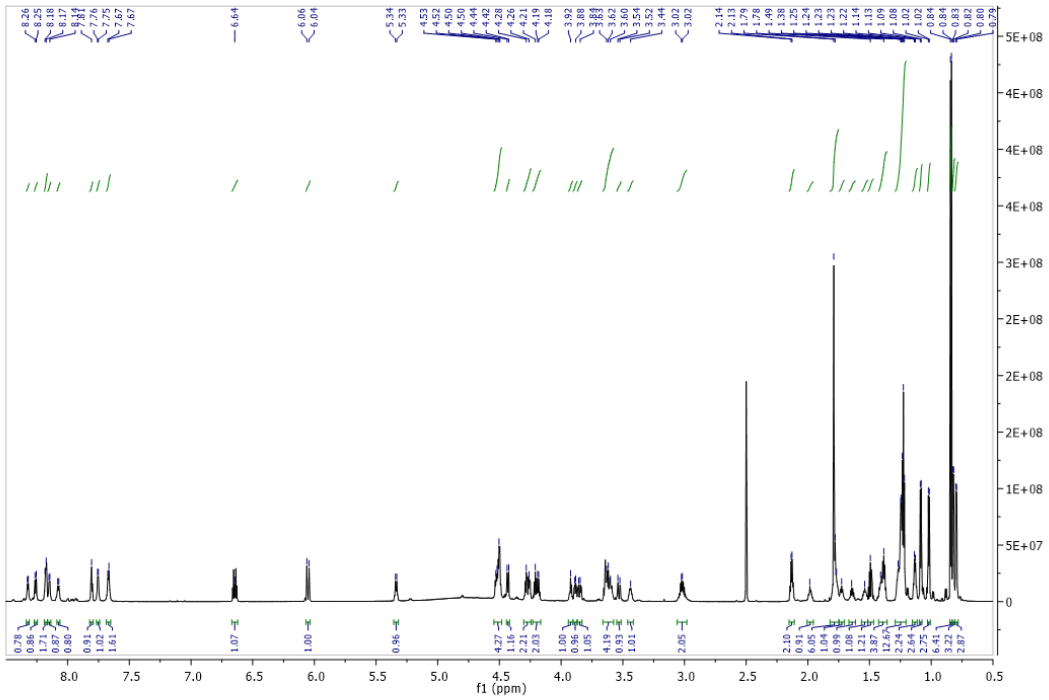

Figure S2. ^{13}C NMR spectrum of taeanamide A (**1**) at 200 MHz in DMSO-d_6.

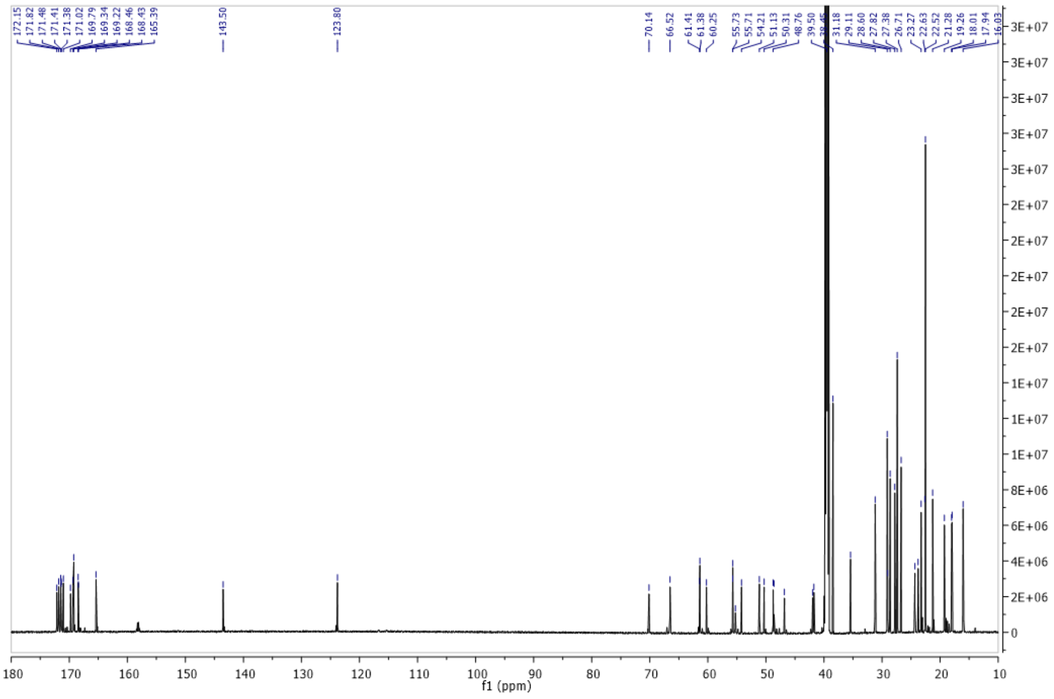

Figure S3. COSY NMR spectrum of taeanamide A (**1**) at 800 MHz in DMSO-d_6.

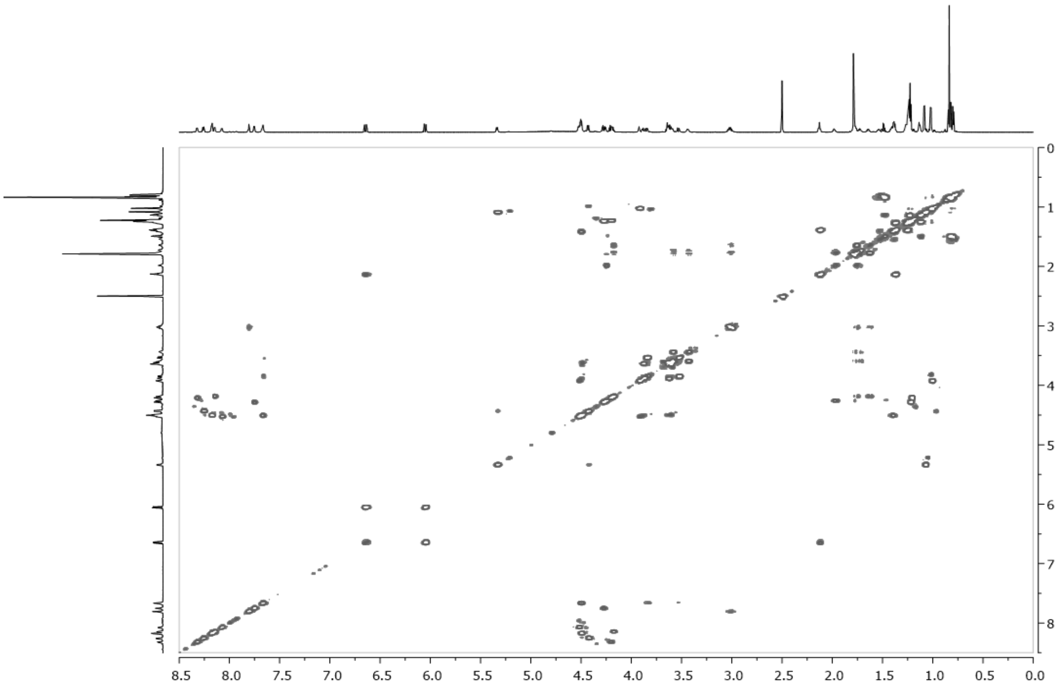

Figure S4. HSQC NMR spectrum of taeanamide A (**1**) at 800 MHz in DMSO-d_6.

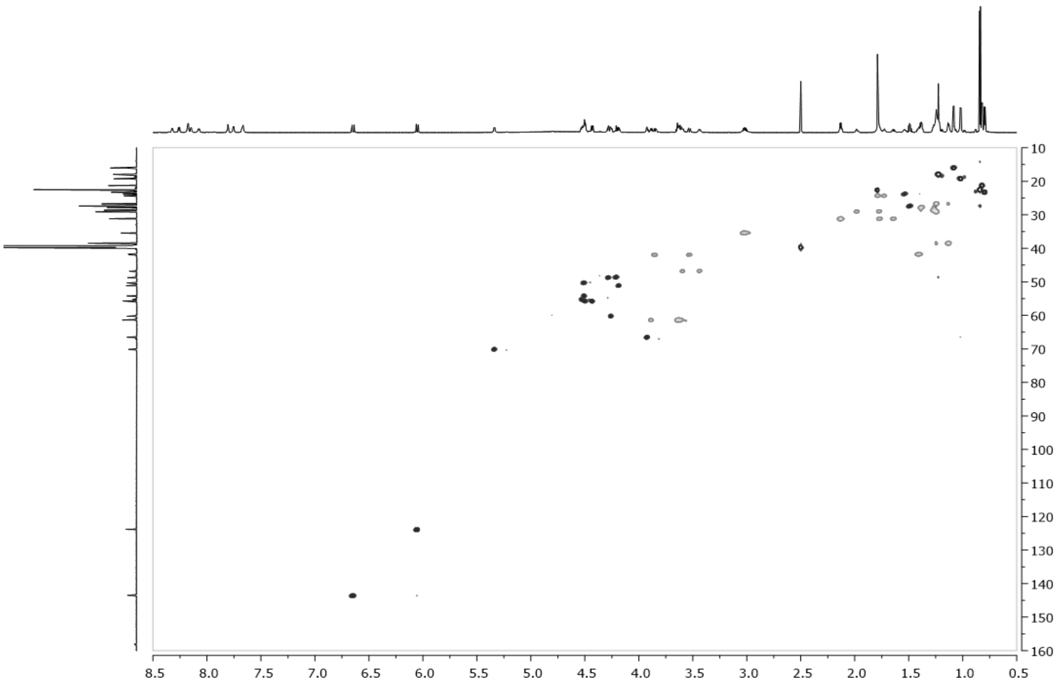

Figure S5. HMBC NMR spectrum of taeanamide A (**1**) at 800 MHz in DMSO-d_6.

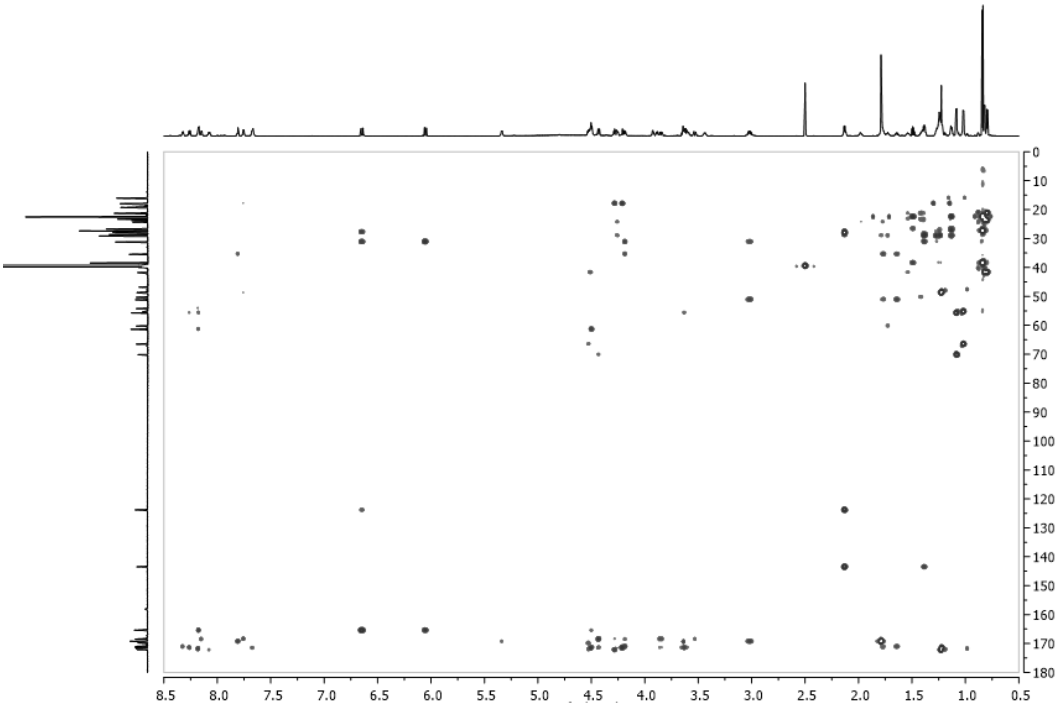

Figure S6. ROESY NMR spectrum of taeanamide A (**1**) at 800 MHz in DMSO-d_6.

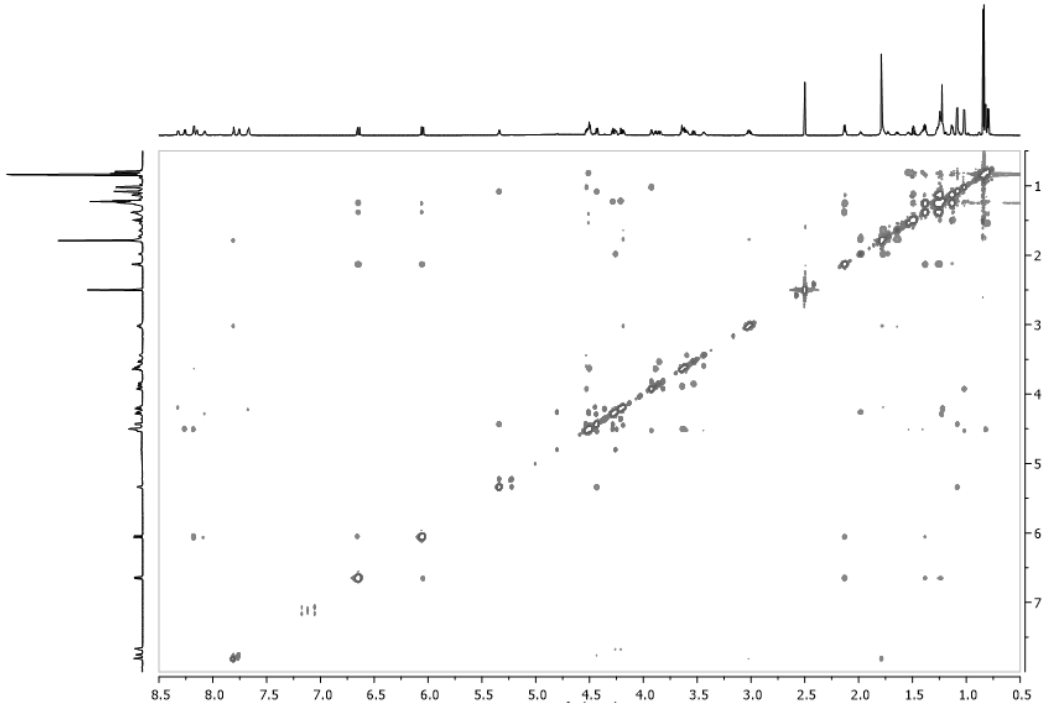

Figure S7. TOCSY NMR spectrum of taeanamide A (**1**) at 800 MHz in DMSO-d_6.

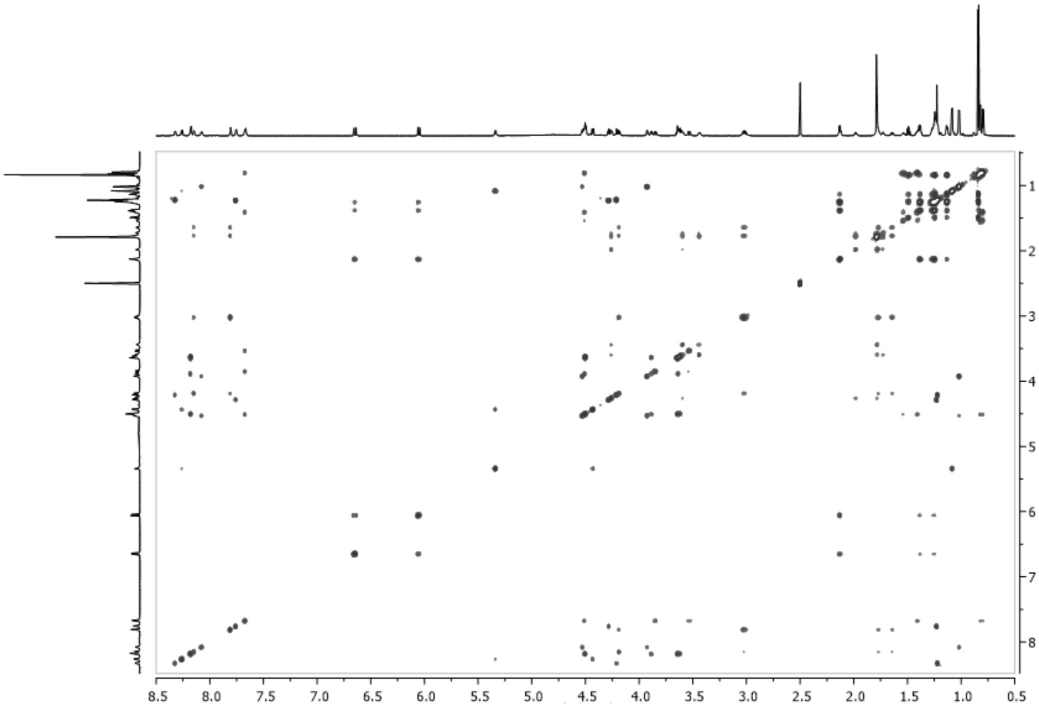

Figure S8. ^{1}H NMR spectrum of taeanamide B (**2**) at 800 MHz in pyridine-d_5.

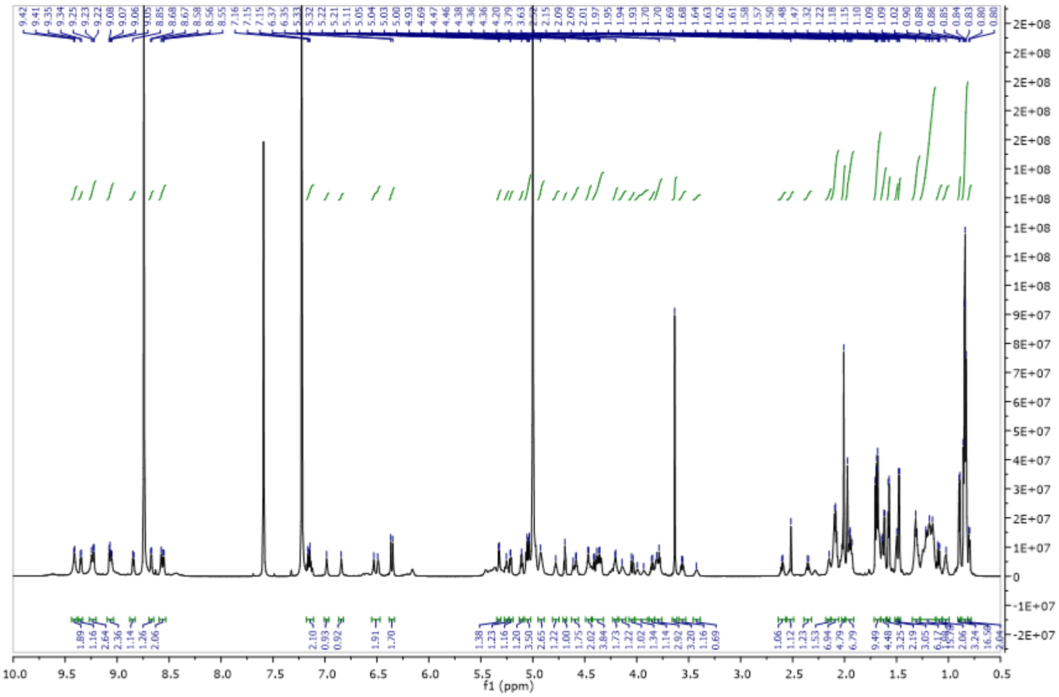

Figure S9. ^{13}C NMR spectrum of taeanamide B (**2**) at 200 MHz in pyridine-d_5.

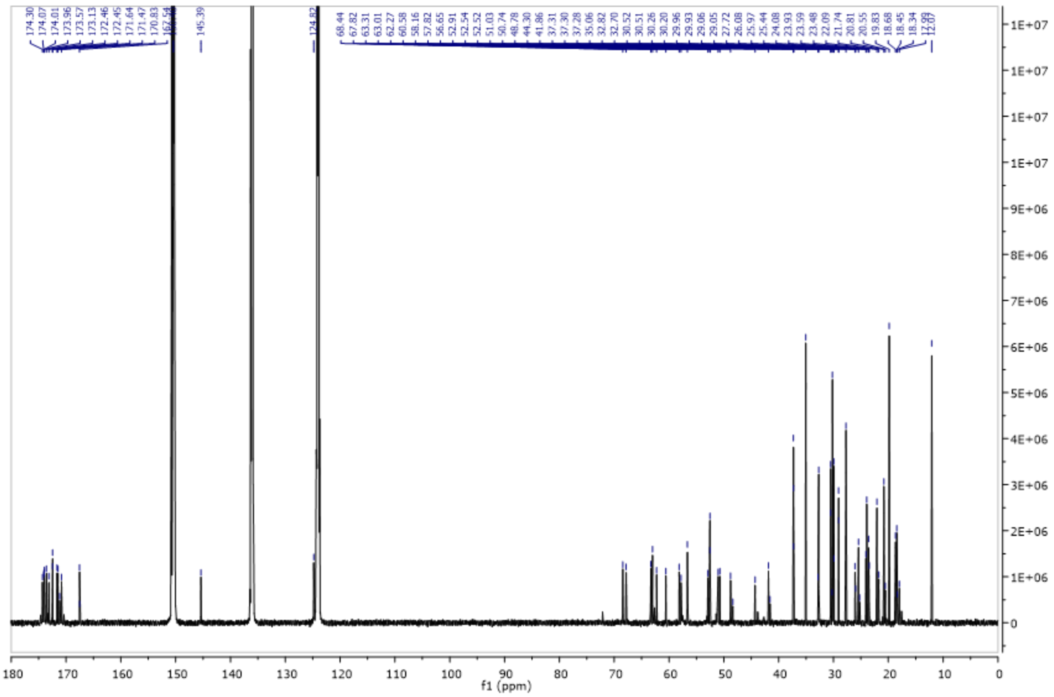

Figure S10. COSY NMR spectrum of taeanamide B (**2**) at 800 MHz in pyridine-d_5.

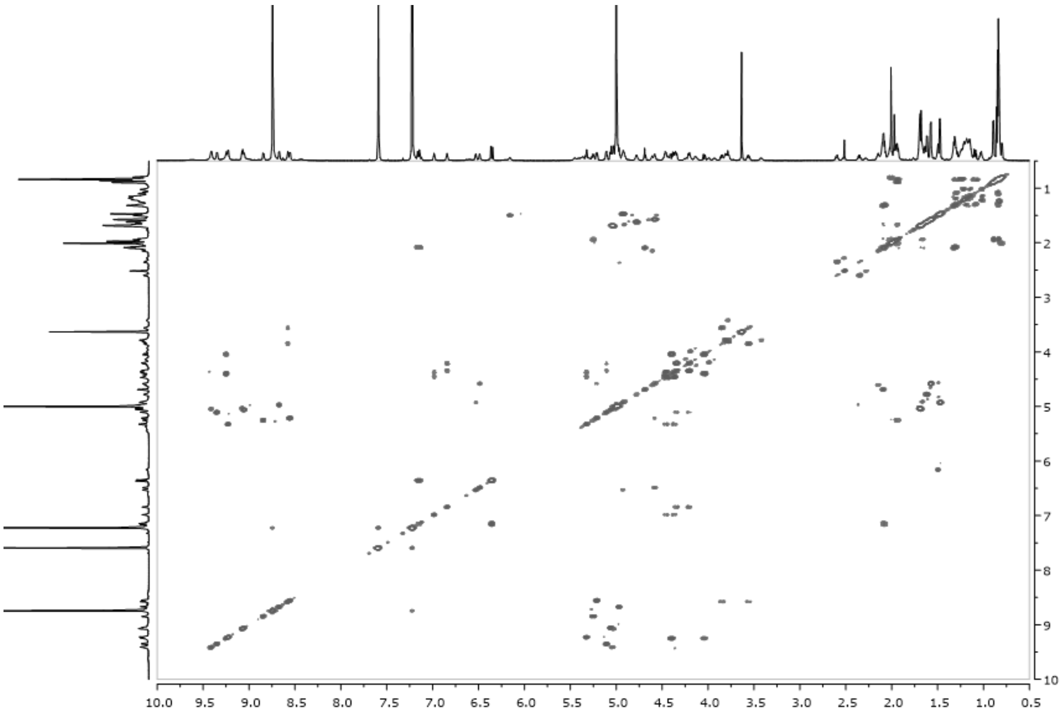

Figure S11. HSQC NMR spectrum of taeanamide B (**2**) at 800 MHz in pyridine-d_5.

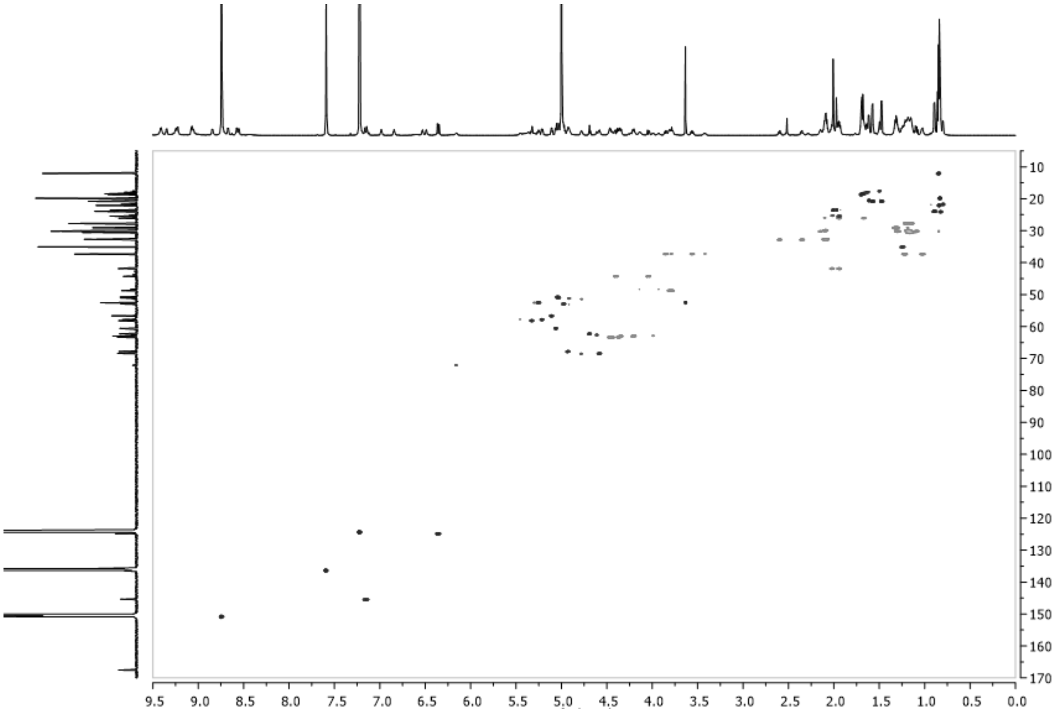

Figure S12. HMBC NMR spectrum of taeanamide B (**2**) at 800 MHz in pyridine-d_5.

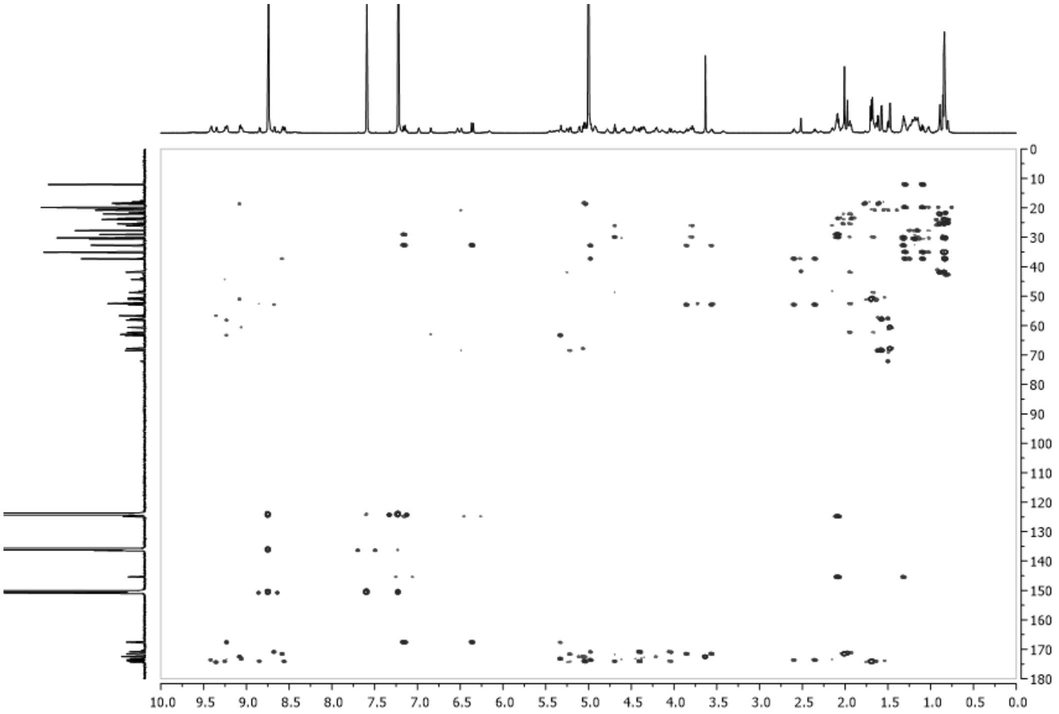

Figure S13. ROESY NMR spectrum of taeanamide B (**2**) at 800 MHz in pyridine-d_5.

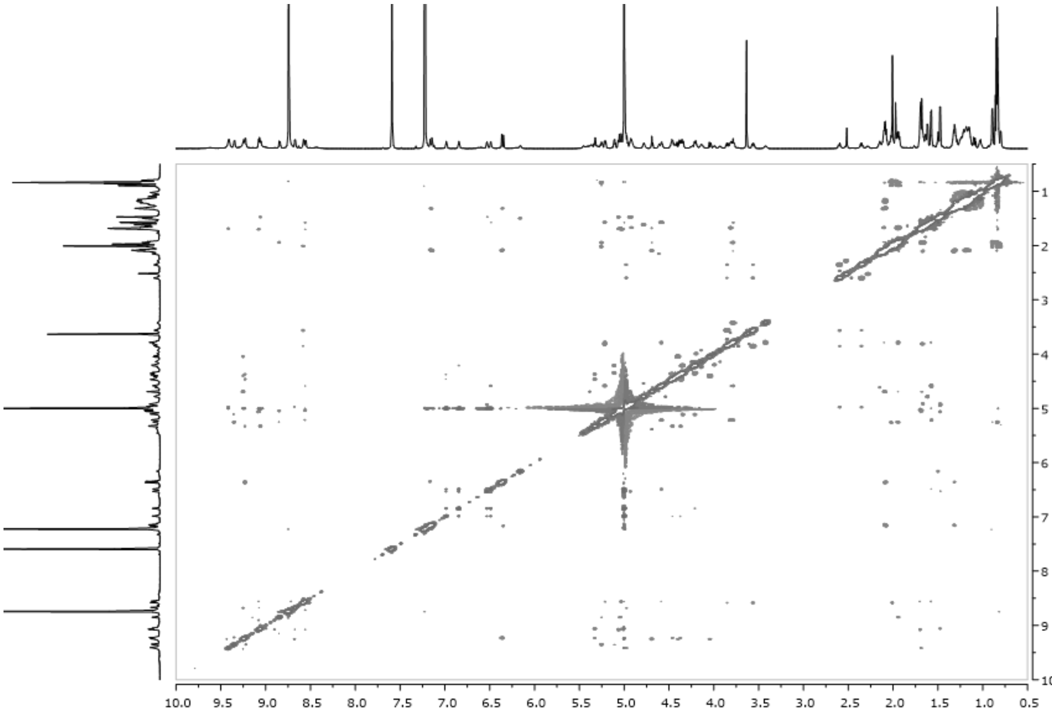

Figure S14. TOCSY NMR spectrum of taeanamide B (**2**) at 800 MHz in pyridine-d_5.

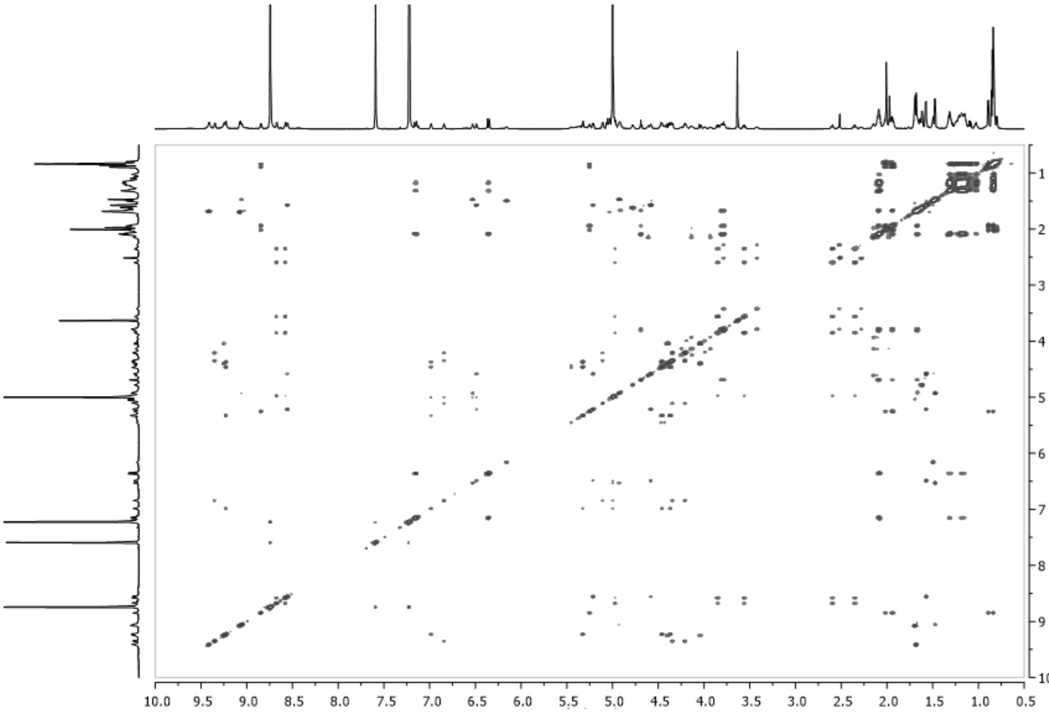

Figure S15. CD spectra of taeanamides A (**1**) and B (**2**).

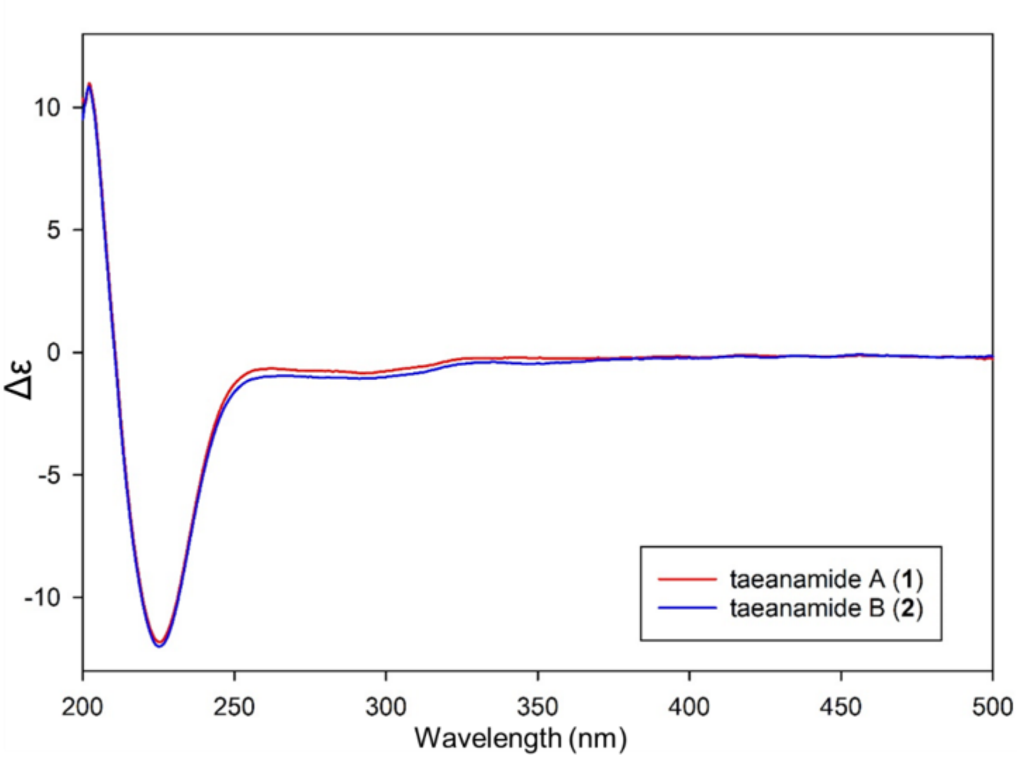

Figure S16. HR-MS/MS data of taeanamide A (**1**) and B (**2**) obtained from a Waters XEVO® G2S Q-TOF mass spectrometer.

(a) HR-MS/MS data of taeanamide A (**1**).

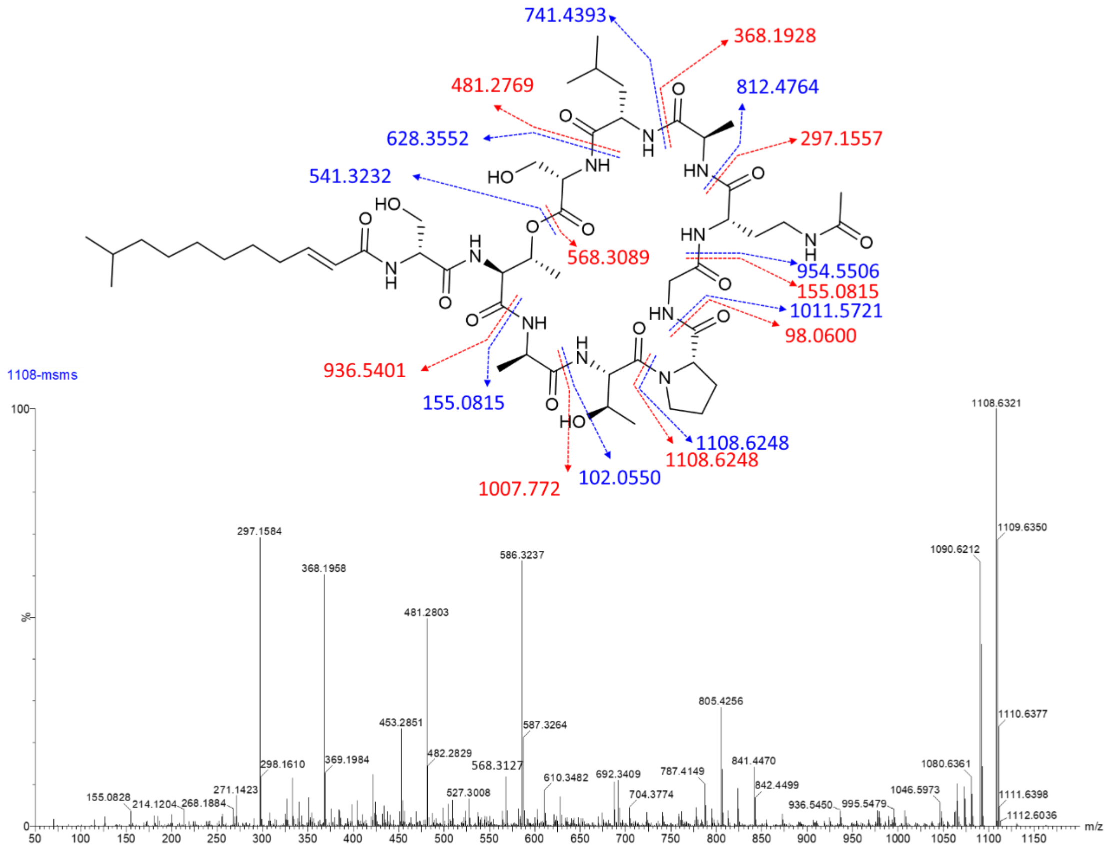

(b) HR-MS/MS data of taeanamide B (**2**).

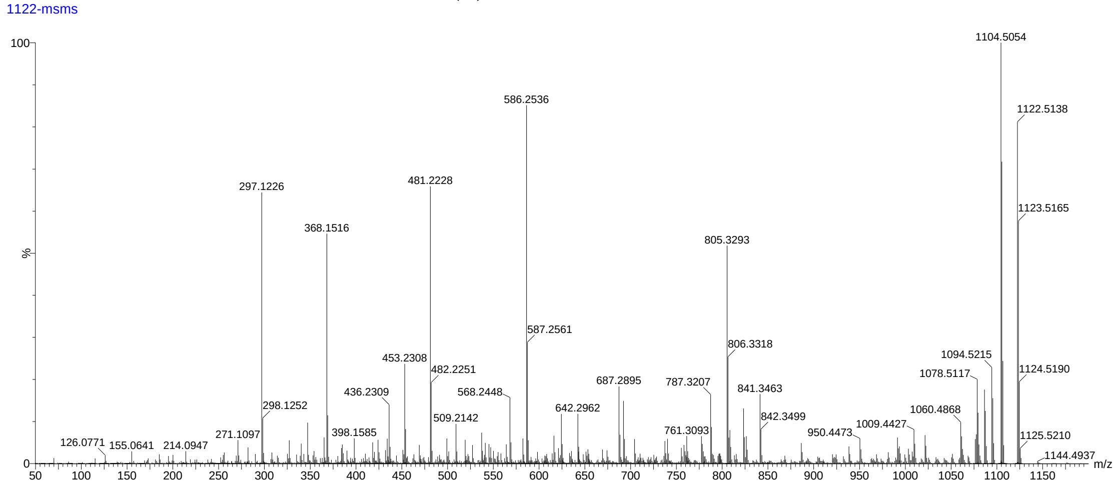

Table S1. LC/MS analysis of L- and D-FDAA derivatives of the amino acids in taeanamide A (**1**).

Taeanamide A						
	Ala	Ser	Thr	Leu	Pro	*N*(4)-Acetyl-2,4-diaminobutyric acid → 2,4-diamino-butanoic acid
$[M+H]^+$ (*m/z*)	342	358	372	384	368	623
Retention time (min)						
L-FDAA	26.5	18.5; 19.0	19.5	34.5	24.5	33.8
D-FDAA	23.6	18.5; 19.0	23.0	38.5	25.8	35.3
Elution order	D→L	N	L→D	L→D	L→D	L→D
Configuration	D	Not determined	L	L	L	L

Table S2. LC/MS analysis of L- and D-FDAA derivatives of the amino acids in taeanamide B (**2**).

Taeanamide B						
	Ala	Ser	Thr	Leu	Pro	*N*(4)-Acetyl-2,4-diaminobutyric acid → 2,4-diamino-butanoic acid
$[M+H]^+$ (*m/z*)	342	358	372	384	368	623
Retention time (min)						
L-FDAA	26.1	17.9; 18.5	19.1	34.0	24.0	33.4
D-FDAA	23.0	18.0; 18.6	22.6	38.1	25.3	35.3
Elution order	D→L	N	L→D	L→D	L→D	L→D
Configuration	D	Not determined	L	L	L	L

Table S3. LC/MS analysis of L- and D-FDAA derivatives of L-2,4-diamino butanoic acid authentic sample.

	L-2,4-diamino butanoic acid authentic sample
+ L-FDAA	33.6
+ D-FDAA	35.3
Elution order	L→D

Table S4. Putative functions of ORFs of the taeanamides biosynthetic gene cluster in *Streptomyces* sp. AMD43.

ORF	Size (aa)	Putative function	Best match	
			Organism/GenBank (residue)	Identity/ Similarity (%)
Orf1	389	Hypothetical protein	*Streptomyces sp.* Ag109_O5-10 / WP_143063641.1 (389)	95/97
Orf2	435	N-6 DNA methylase	*Streptomyces flavoviridis* / WP_189596001.1 (435)	98/98
TaemA	4512	NRPS		
TaemB	486	Diaminobutyrate aminotransferase	*Streptomyces sp.* SAI-083 / WP_123988349.1 (423)	91/95
TaemC	265	Thioesterase	*Streptomyces sp.* S063 / WP_128818897.1 (256)	56/70
TaemD	70	MbtH protein	*Streptomyces chrestomyceticus* / WP_125043567.1 (76)	67/84
Orf3	92	Transposase	*Streptomyces sp.* Ag82_G6-1 / WP_097221223.1 (383)	75/86
Orf4	85	Transposase	*Streptomyces sp.* CB01580 / OKJ25938.1 (241)	75/79
Orf5	187	Transposase	*Streptomyces sp.* Ag109_O5-1 / WP_123981086.1 (374)	81/82
Orf6	725	Integral membrane protein	*Streptomyces tsukubensis* / WP_077968499.1 (736)	62/73
Orf7	67			
TaemE	3786	NRPS		
TaemF	3211	NRPS		
Orf8	394	Maturase	*Streptomyces sp.* F001 / WP_129803435.1 (608)	83/89
Orf9	570	Recombinase family protein	*Streptomyces sp.* AS58/ WP_079001718.1 (641)	97/98

Table S5. Adenylation (A) domain substrate specificity predictions of the taeanamide NRPS. The underlined amino acids in the substrate specific code are residues that do not match the Stachelhaus codes [1].

A domain	Substrate specific code	Stachelhaus code match	Predicted amino acid	Actual amino acid in taeanamide
A1	D V W H I S L V D K	100%	Ser	D-Ser
A2	D F W S V G M V H K	100%	Thr	L-Thr
A3	D V R H I S L V E K	80%	Ser	D-Ala
A4	D F W S V G M V H K	100%	Thr	L-Thr
A5	D V Q Y A A H V V K	90%	Pro	L-Pro
A6	D I L Q L G L I W K	100%	Gly	Gly
A7	D A R Q I G L V D K	70%	Gln	*N*-Ac-L-Dab
A8	D V R H I S L V E K	80%	Ser	D-Ala
A9	D A L L I G A V A K	80%	Val	L-Leu
A10	D V W H I S L V D K	100%	Ser	L-Ser

Figure S17. Multiple sequence alignment of condensation (C) domains of Taem NRPS and lipopeptide synthetases. Blue boxes indicate core motifs 1 through 5 of C domains [2]. It includes specific conserved residues at each motif according to C domain subtype. E/C domains contain a characteristic HH[I/L]xxxxGD motif at their N-terminus (Red box). Amino acid sequences of comparative C domains are extracted from daptomycin synthetase (*Streptomyces filamentosus* NRRL 11379, GenBank AY787762), surfactin synthetase (*Bacillus velezensis* FZB42, GenBank AJ575642), arthrofactin synthetase (*Pseudomonas* sp. MIS38, GenBank AB107223), fusaricidin synthetase (*Paenibacillus polymyxa*, GenBank EF451155), and syringafactin synthetase (*Pseudomonas syringae* pv. tomato str. DC3000, GenBank AE016853).

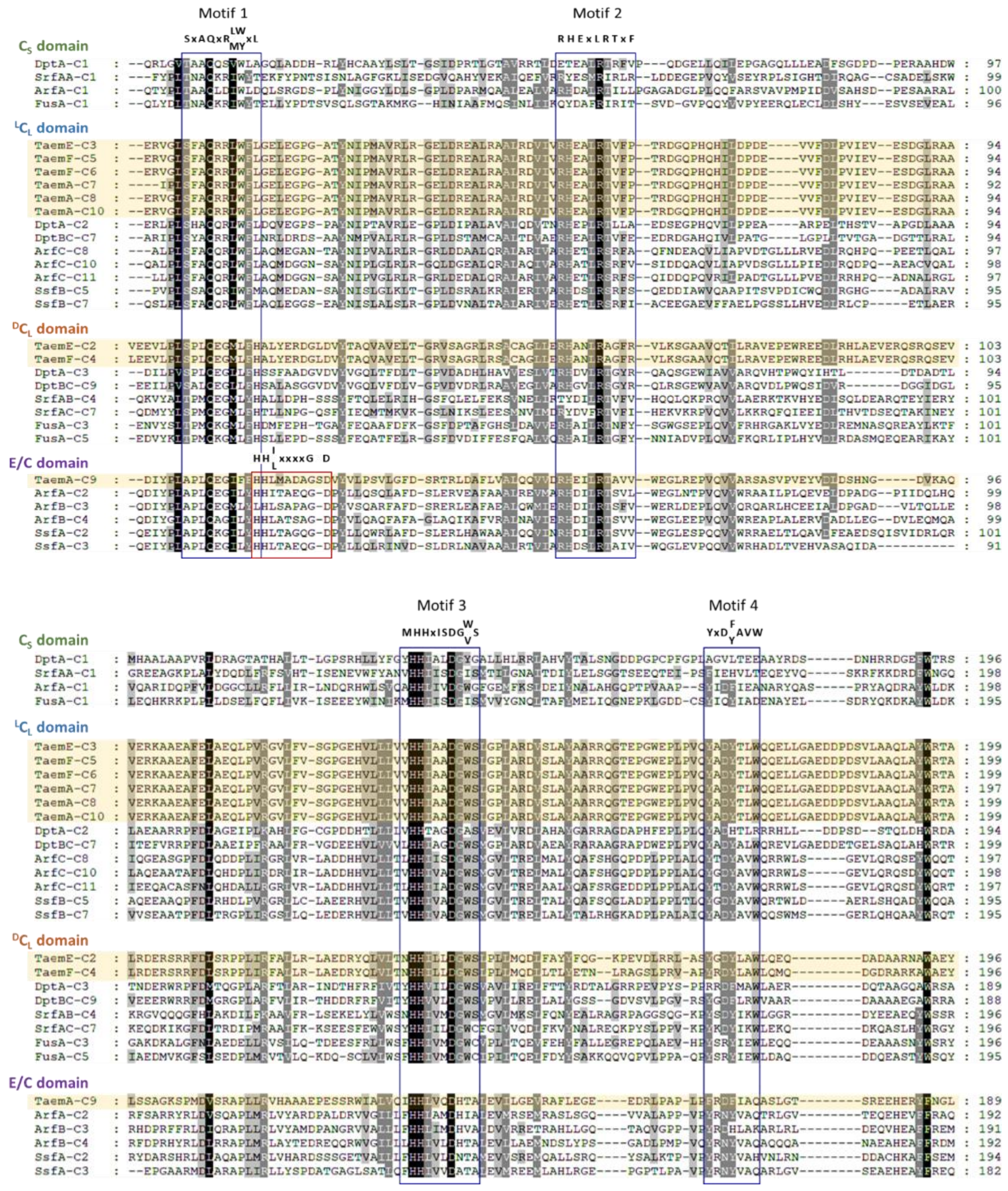

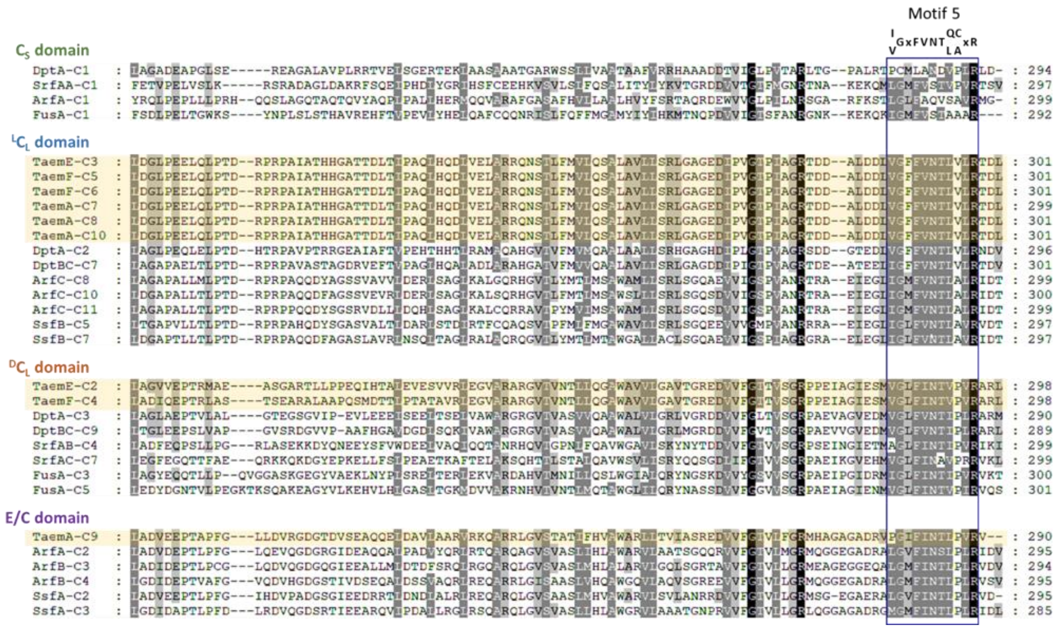
Motif 5
C$_S$ domain
DptA-C1
SrfAA-C1
ArfA-C1
FusA-C1
^{L}C$_L$ domain
TaemE-C3
TaemF-C5
TaemF-C6
TaemA-C7
TaemA-C8
TaemA-C10
DptA-C2
DptBC-C7
ArfC-C8
ArfC-C10
ArfC-C11
SsfB-C5
SsfB-C7
^{D}C$_L$ domain
TaemE-C2
TaemF-C4
DptA-C3
DptBC-C9
SrfAB-C4
SrfAC-C7
FusA-C3
FusA-C5
E/C domain
TaemA-C9
ArfA-C2
ArfB-C3
ArfB-C4
SsfA-C2
SsfA-C3

Figure S18. Phylogenetic analysis of condensation (C) domains of Taem NRPS and lipopeptide synthetases. Amino acid sequences of comparative C domains are extracted from daptomycin synthetase (*Streptomyces filamentosus* NRRL 11379, GenBank AY787762), surfactin synthetase (*Bacillus velezensis* FZB42, GenBank AJ575642), arthrofactin synthetase (*Pseudomonas* sp. MIS38, GenBank AB107223), fusaricidin synthetase (*Paenibacillus polymyxa*, GenBank EF451155), syringafactin synthetase (*Pseudomonas syringae* pv. tomato str. DC3000, GenBank AE016853), and CDA synthetase (*Streptomyces coelicolor* A3(2), GenBank AL645882).

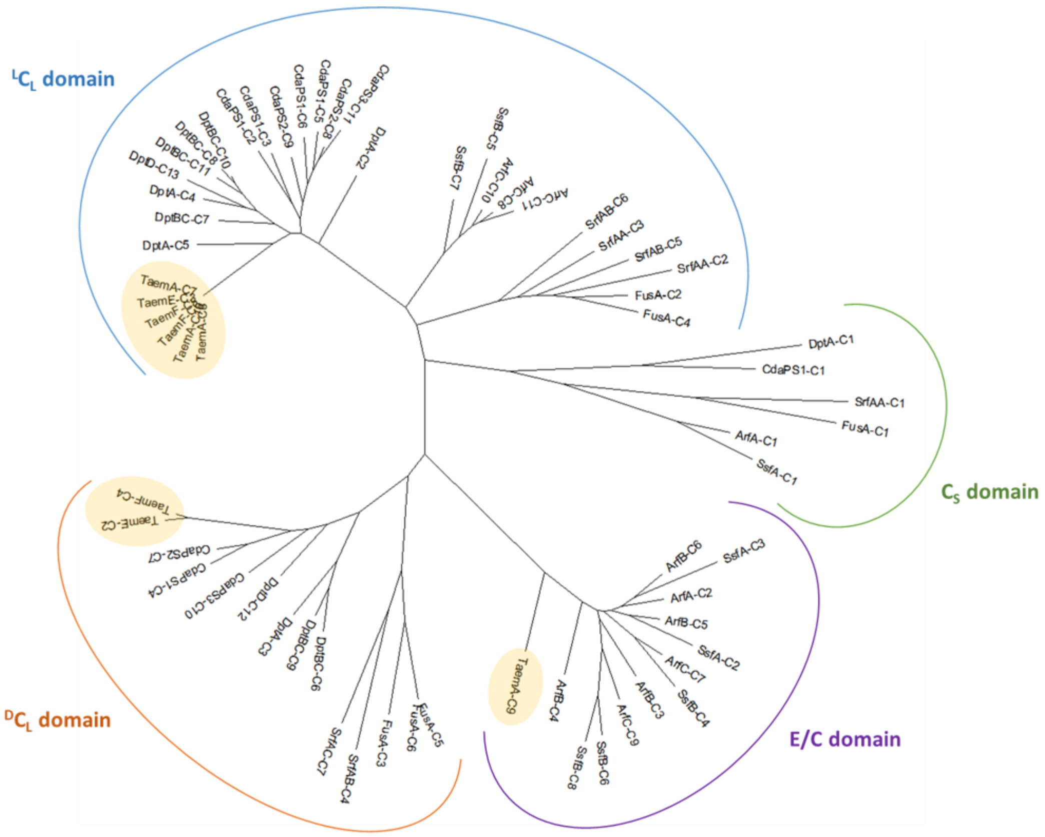

Figures S19. LC/MS profiles of (a) EtOAc extract of strain AMD43 showing the existence of both taeanamides A and B (**1** and **2**); (b) taeanamide A (**1**) after purification; (c) taeanamide A (**1**) after 10 days in MeOH (room temperature).

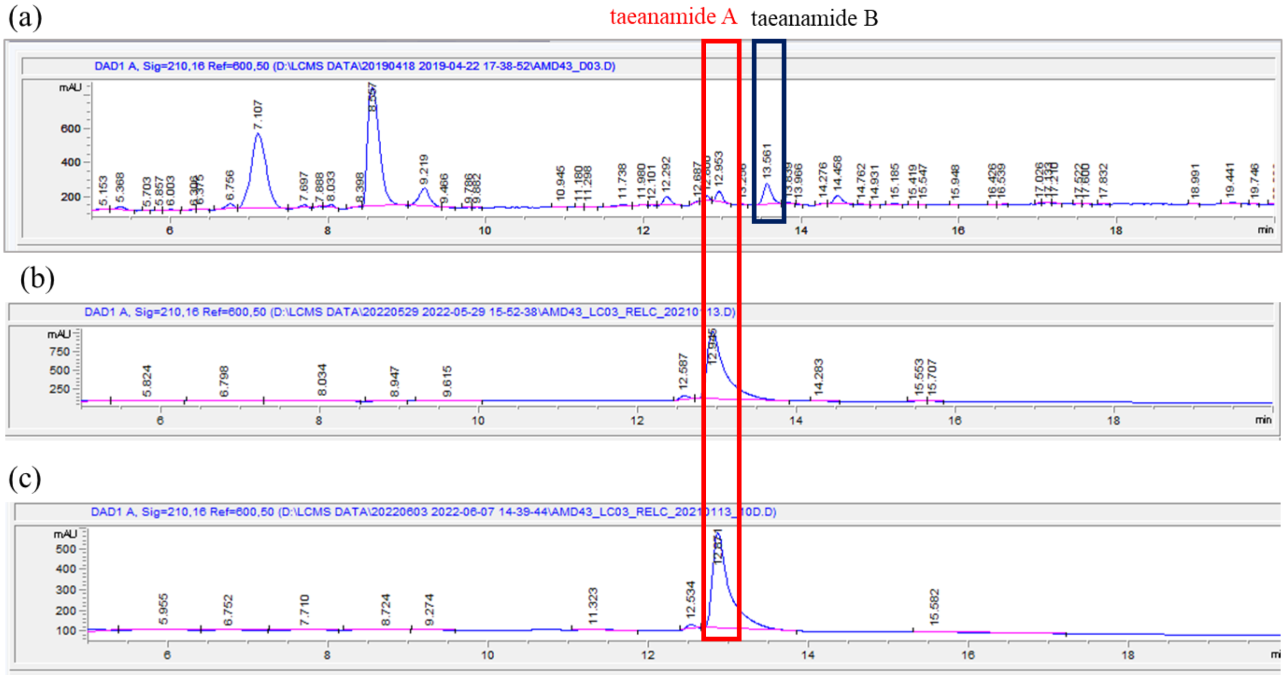

References

1. Stachelhaus, T.; Mootz, H. D.; Marahiel, M. A. The specificity-conferring code of adenylation domains in nonribosomal peptide synthetases. *Chem. Boil.* **1999**, 6, 493-505.
2. Rausch et al. Phylogenetic analysis of condensation domains in NRPS sheds light on their functional evolution. *BMC. Evol. Biol.* **2007**, 7, 78.
